# Single-Cell RNA Sequencing Analysis Reveals Greater Epithelial Ridge Cells Degeneration During Postnatal Development of Cochlea in Rats

**DOI:** 10.3389/fcell.2021.719491

**Published:** 2021-09-03

**Authors:** Jianyong Chen, Dekun Gao, Junmin Chen, Shule Hou, Baihui He, Yue Li, Shuna Li, Fan Zhang, Xiayu Sun, Fabio Mammano, Lianhua Sun, Jun Yang, Guiliang Zheng

**Affiliations:** ^1^Department of Otorhinolaryngology Head and Neck Surgery, Xinhua Hospital, Shanghai Jiao Tong University School of Medicine, Shanghai, China; ^2^Shanghai Jiao Tong University School of Medicine Ear Institute, Shanghai, China; ^3^Shanghai Key Laboratory of Translational Medicine on Ear and Nose Diseases, Shanghai, China; ^4^Department of Physics and Astronomy “G. Galilei”, University of Padova, Padua, Italy; ^5^Department of Biomedical Sciences, Institute of Cell Biology and Neurobiology, Italian National Research Council, Monterotondo, Italy

**Keywords:** single-cell RNA sequencing, cochlear duct, greater epithelial ridge cell, landscape, degeneration

## Abstract

Greater epithelial ridge cells, a transient neonatal cell group in the cochlear duct, which plays a crucial role in the functional maturation of hair cell, structural development of tectorial membrane, and refinement of audio localization before hearing. Greater epithelial ridge cells are methodologically homogeneous, while whether different cell subtypes are existence in this intriguing region and the degeneration mechanism during postnatal cochlear development are poorly understood. In the present study, single-cell RNA sequencing was performed on the cochlear duct of postnatal rats at day 1 (P1) and day 7 (P7) to identify subsets of greater epithelial ridge cell and progression. Gene ontology and Kyoto Encyclopedia of Genes and Genomes pathway enrichment analysis were used to examine genes enriched biological processes in these clusters. We identified a total of 26 clusters at P1 and P7 rats and found that the cell number of five cell clusters decreased significantly, while four clusters had similar gene expression patterns and biological properties. The genes of these four cell populations were mainly enriched in Ribosome and P13K-Akt signal pathway. Among them, Rps16, Rpsa, Col4a2, Col6a2, Ctsk, and Jun are particularly interesting as their expression might contribute to the greater epithelial ridge cells degeneration. In conclusion, our study provides an important reference resource of greater epithelial ridge cells landscape and mechanism insights for further understanding greater epithelial ridge cells degeneration during postnatal rat cochlear development.

## Introduction

Mammalian hearing is dependent on the normal development of the cochlea, which includes the development of hair cells and supporting cells. The main cause of sensorineural hearing loss is damage to hair cells, which is difficult to reverse after hearing development has matured (Wagner and Shin, 2019). It has been found that hair cells in mice have a transient and limited ability to regenerate during hearing development, and further studies have revealed that this is related to the unique structural components of the inner ear (Henley et al., 1996; Chen et al., 2019).

The mammalian inner ear contains the vestibular sensory epithelium that perceives various accelerations and the auditory epithelia that perceive sound stimuli (Kelley, 2007). The auditory epithelium contains two main cell types: supporting cells and hair cells with greatly different anatomical and physiological characteristics (Scheffer et al., 2015). Hair cells continuously receive various stimuli from supporting cells and the outside world through unique receptors during their growth and development. These include the G-protein coupled P2Y receptors that activate phospholipase C (PLC) dependent production of inositor triphosphate (IP3) and release of intracellular ionized calcium (Ca^2 +^) from intracellular stores (Forsythe, 2007; Tritsch et al., 2007). In the developing cochlea, glutamate release from IHCs activates type I spiral neurons (SGNs) to generate action potentials, thereby mimicking in the pre-hearing cochlea the mechanical-electrical signal transduction effect triggered by acoustic waves transmitted via the external auditory canal (Tritsch and Bergles, 2010; Mammano and Bortolozzi, 2018; Ceriani et al., 2019), resulting in an increase in the frequency of spontaneous action potential release from IHCs and promoting the functional maturation of IHCs (Flores-Otero et al., 2007; Tritsch and Bergles, 2010; Dayaratne et al., 2014).

There are various types of supporting cells in the cochlea, and our group’s previous research focused on GER cells. We found direct evidence of ATP release from supporting cells, with interactions between proteins involved in the ATP synthesis, release and action pathway in the cochlea, and the intracellular ATP-containing vesicles are lysosomes (He and Yang, 2015a). In addition, we found that not only apoptosis but also autophagy of the supporting cells occurs in GER (Yang and He, 2016; Hou et al., 2020). The GER cells are a group of broad columnar support cells located medial to the hair cells in the cochlea, and is the earliest epithelial structure to appear during cochlear development (Lim and Anniko, 1985). GER cells stimulate the production of calcium waves in the supporting cells by spontaneously releasing ATP, which excites hair cells and afferent nerve fibers, triggers the development of action potentials in auditory nerve fibers, and synchronizes the transmitters released by IHCs to encode similar frequencies, playing an important role in the development of the cochlea (Mazzarda et al., 2020). GER cells are seen in numerous mammals, including human, rat, and mouse, and undergo a series of changes in cell morphology and number during embryonic and postnatal periods (Hinojosa, 1977; Lim and Anniko, 1985), such as cell cytoplasm moving away from the cytosol, cell crumpling, gradual increase in cell gaps, gradual replacement of columnar cells by cuboidal cells, etc., eventually degenerating and leading to a mature inner sulcus region on the Corti organ structure after the animal becomes sensitive to external acoustic stimuli (Kelley, 2007; Nickel and Forge, 2008; Dayaratne et al., 2014). Eventually, the GER cells disappear while cochlear development continues, leading to a fully mature auditory function.

Transcriptome sequencing has been extensively used in previous studies of the inner ear (Liu et al., 2014; Burns et al., 2015; Cai et al., 2015; Scheffer et al., 2015; Han et al., 2018; Cheng et al., 2019; Tang et al., 2019; Li et al., 2020). However, it remains to be established whether GER cells degenerate completely during development or are partially transformed into other cell types to achieve a mature auditory function. To examine the transcriptional changes that occur during the formation of the organ of Corti, Kolla et al. (2020) performed single-cell RNA sequencing of the cochlear basilar membrane at different developmental stages and clustered them according to different gene expression patterns. Kolla’s research team dissociated cochlear duct cells at four developmental time points and captured individual cells for analysis using single-cell RNAseq. They identified multiple unique cell types at each time point, including both known types, such as HCs and SCs, and previously unknown cell types, such as multiple unique cell types in Kölliker’s organ. The sampling in the Kolla’s team study was from E14 and E16 mice, whereas here we focussed our attention on rats at P1 and P7. However, the molecular mechanisms of how these cell clusters regulate GER cells development through changes in gene expression patterns at different times are not clear. Molecular signals released by GER cells during different periods may also shape hair cell maturation and auditory development by driving gene transcription, altering GER cell morphology, and changes in the number of GER cells, all of which are closely associated with hair cell maturation (Uziel, 1986; Legrand et al., 1988).

In the present work, we revealed the changes of GER cells profiles by single-cell RNA sequencing technology (Baslan et al., 2012; Shapiro et al., 2013; Huang et al., 2018). We also observed the change of the expression and regulation of key genes and signaling pathways enriched biological processes in these clusters during cochlear development. We identified four clusters with similar gene expression patterns and biological properties. Further investigations showed the genes of these four cell clusters were mainly enriched in Ribosome and P13K-Akt signal pathway. Among them, Rps16, Rpsa, Col4a2, Col6a2, Ctsk, and Jun are particularly interesting as their expression might contribute to degeneration of GER cells during normal development. Our present data provide a reference for the cellular landscape of the GER and suggests possible mechanism that lead to GER degeneration during normal postnatal development.

## Materials and Methods

### Animals

Male and/or female Sprague-Dawley (SD) rats were purchased from Shanghai SIPPR-BK Laboratory Animals Co. Ltd. All the procedures involving rats were performed following the guidelines approved by the institutional Animal Care and Use Committee of the Shanghai Jiao Tong University School of Medicine.

In this study, the first postnatal day (P1) was the birthday, and P7 means the postnatal time points after the birthday. Forty SD rats were randomly selected for each age group. The rats were sacrificed using an approved guillotine method. The cochlea tissue was extracted from the temporal bone, and the otic capsule was carefully transferred to a dish containing 0.01 M cold sodium phosphate-buffered saline (PBS, pH 7.35, GIBCO, Invitrogen Inc., Carlsbad, CA, United States). The stria vascularis, spiral ligament, and spiral ganglions, was gently separated by microdissection. The isolated cochlear duct was washed twice with potassium and magnesium-free PBS.

### Tissue Dissociation and Preparation of Single-Cell Suspensions

The cochlear duct tissues were placed in a sterile RNase-free culture dish containing an appropriate amount of calcium-free and magnesium-free 1 × PBS on ice, the tissues were transferred into the culture dish and cut it into 0.5 mm^2^ pieces, the tissues were washed with 1 × PBS, and remove as many non-purpose tissues as possible such as blood stains and fatty layers. Tissues were dissociated into single cells in dissociation solution (0.35% collagenase IV5, 2 mg/ml papain, 120 Units/ml DNase I) in 37°C water bath with shaking for 20 min at 100 rpm. Digestion was terminated with 1 × PBS containing 10% fetal bovine serum (FBS, V/V, then pipetting 5–10 times with a Pasteur pipette). The resulting cell suspension was filtered by passing through a 70–30 μm stacked cell strainer and centrifuged at 300 *g* for 5 min at 4°C. The cell pellet was resuspended in 100 μl 1 × PBS (0.04% BSA) and added with 1 ml 1 × red blood cell lysis buffer (MACS 130-094-183, 10×)and incubated at room temperature or on ice for 2–10 min to lyse remaining red blood cells.

After incubation, the suspension was centrifuged at 300 *g* for 5 min at room temperature. The suspension was resuspended in 100 μl Dead Cell Removal MicroBeads (MACS 130-090-101) and remove dead cells using Miltenyi Dead Cell Removal Kit (MACS 130-090-101). Then the suspension was resuspended in 1 × PBS(0.04% BSA) and centrifuged at 300 *g* for 3 min at 4°C (repeat twice). The cell pellet was resuspended in 50 μl of 1 × PBS (0.04% BSA). The overall cell viability was confirmed by trypan blue exclusion, which needed to be above 85%, single-cell suspensions were counted using a hemocytometer/Countess II Automated Cell Counter and concentration adjusted to 700–1200 cells/μl.

### Chromium 10x Genomics Library and Sequencing

Single-cell suspensions were loaded to 10x Chromium to capture 5000 single cells according to the manufacturer’s instructions of the 10X Genomics Chromium Single-Cell 3′ kit (V3). The following cDNA amplification and library construction steps were performed according to the standard protocol. Libraries were sequenced on an Illumina NovaSeq 6000 sequencing system (paired-end multiplexing run, 150 bp) by LC-Bio Technology Co. Ltd. (Hangzhou, China) at a minimum depth of 20,000 reads per cell.

### Bioinformatics Analysis of scRNA-Seq Data

Sequencing results were demultiplexed and converted to FASTQ format using Illumina bcl2fastq software. Sample demultiplexing, barcode processing, and single-cell 3′ gene counting by using the Cell Ranger pipeline^1^ (version 3.1.0) and scRNA-seq data were aligned to Rattus norvegicus reference genome (Source: Rattus norvegicus UCSC; version: rn6). The Cell Ranger output was loaded into Seurat (version 3.1.1) to be used for dimensional reduction, clustering, and analysis of scRNA-sequencing data. Overall, 25139 cells passed the quality control threshold: all genes expressed in less than 1 cell were removed, the number of genes expressed per cell > 500 as low and <5000 as high cut-off, and UMI counts less than 500 and the percent of mitochondrial-DNA derived gene-expression < 25%.

To visualize the data, we further reduced the dimensionality of all 25139 cells using Seurat and used t-SNE to project the cells into 2D space (Satija et al., 2015), the steps include: (1) Using the Log normalize method of the “Normalization” function of the Seurat software to calculate the expression value of genes; (2) PCA (Principal component analysis) analysis was performed using the normalized expression value, Within all the PCs, the top 10 PCs were used to do clustering and t-SNE analysis; (3) To find clusters, selecting weighted Shared Nearest Neighbor (SNN) graph-based clustering method. Marker genes for each cluster were identified with the Wilcoxon rank-sum test (default parameters is “bi-mod”: Likelihood-ratio test)with default parameters via the “Find All Markers” function in Seurat. This selects markers genes that are expressed in more than 10% of the cells in a cluster and average log_2_ (Fold Change) of greater than 0.26.

### Pathway Enrichment Analysis

Gene ontology (GO) enrichment analyses were performed with top GO package in R (Bioconductor) (Brionne et al., 2019) and Kyoto Encyclopedia of Genes and Genomes (KEGG) pathway enrichment analysis examining enriched processes in clusters was performed using Ingenuity Pathway Analysis (IPA) (Krämer et al., 2014). These two Functional enrichment analyses were used to identify which DEGs were significantly enriched in GO terms and/or metabolic pathways. GO is an international standard classification system for gene function. DEGs are mapped to the GO terms (biological functions) in the database. The number of genes in each term was calculated, and a hypergeometric test was performed to identify significantly enriched GO terms in the gene list out of the background of the reference gene list. GO terms and KEGG pathways with the adjusted *p*-value < 0.05 were considered significantly different.

### Localization of Gene Expression by Fluorescence *in situ* Hybridization (FISH)

The Paraffin-DIG (digoxigenin)-TSA (Tyramide Signal Amplification)-ISH protocol was used to verify the localization of gene expression. Cochleae were collected from SD rats of both sexes at postnatal day 1 (P1) and day 7 (P7) fixed in 4% paraformaldehyde overnight. The tissue was then dehydrated by gradient alcohol, paraffin, embedding, and sectioned on a cryostat at 10-μm thickness. Hybridization protocol was carried out based on the manufacturer’s suggestions. After RNA ISH, sections were washed in 2 × SSC for 10 min at 37°C, with 1 × SSC two times for 5 min at 37°C, and wash in 0.5 × SSC for 10 min at room temperature. Formamide washing can be added if there are more non-specific hybrids. Blocking solution (rabbit serum) was added to the section and incubate at room temperature for 30 min, and then remove the blocking solution and add anti-digoxigenin-labeled peroxidase. Sections were incubated with secondary antibodies at 37°C for 40 min, then washed with PBS four times for 5 min each. And Nuclei were counterstained with DAPI for 15 s at room temperature. All fluorescent images were obtained on a Nikon Eclipse CI upright fluorescence microscope.

### Statistical Analysis

All the statistical analyses of the cochlear cells data described in this paper were performed using Prism version 7.0 (GraphPad Software) and calculated according to the relative abundances. Experimental data are presented as the mean ± standard error of the mean. Comparisons were made by one-way analyses of variance or student’s unpaired two-tailed *t*-tests between two different stages. *P*-values < 0.05 were considered statistically significant differences between these two periods.

## Results

### scRNA-seq Identifies Multiple 26 Clusters of Cochlear Cells at P1 and P7

We performed scRNA-seq using a 10 × Genomics platform in pooled cochlear cells from 40 cochlear tissues from P1 and P7 phases. The cochlear duct cells comprise a highly diverse cellular mosaic that includes an undefined number of supporting cell (SC) types, two different types of mechanosensory hair cells (HCs), and unknown cell types in Kölliker’s organ (KO).

**FIGURE 1:**
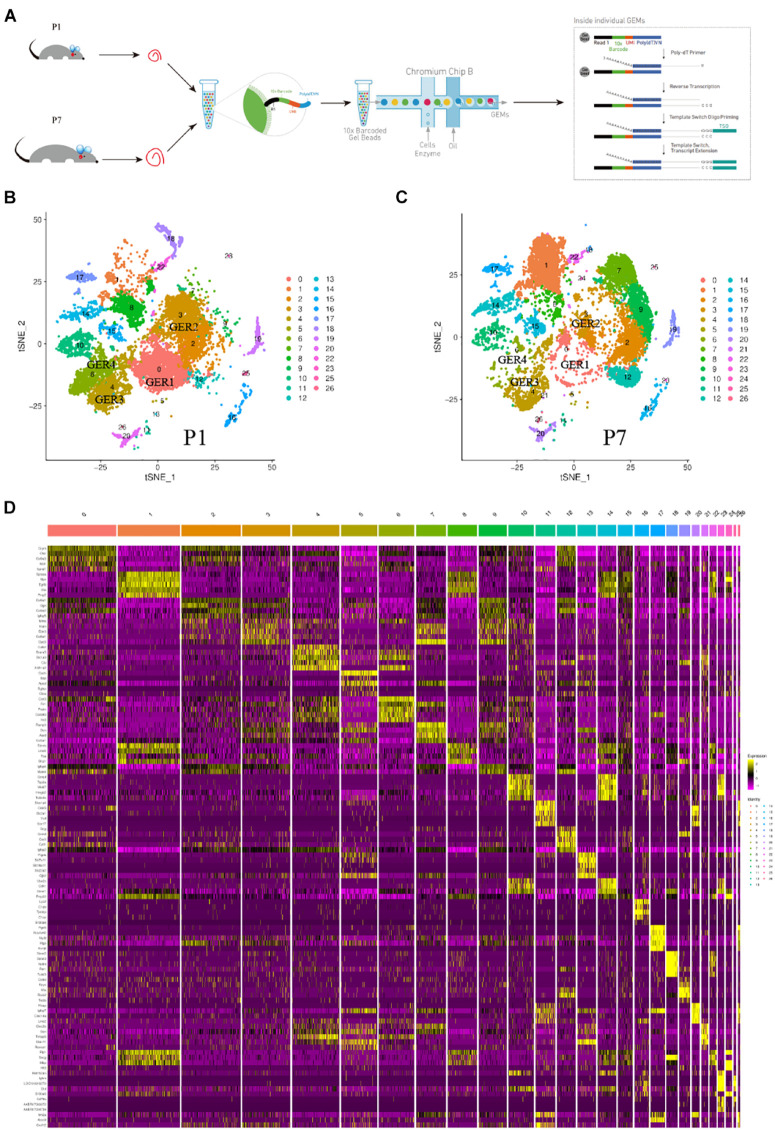
Global expression profiling of cochlear cells by scRNA-Seq and cell clusters identification. **(A)** Scheme of cochlear duct preparation, single-cell isolation, and Chromium 10x Genomics library and scRNA-seq at P1 and P7. **(B)** t-SNE plots of cochlear cells at P1. (Left) Cell colors based on the origins (donors) are shown. **(C)** t-SNE plots of cochlear cells at P7. (Right) Cell colors based on the origins (donors) are shown. **(D)** Heat map for cochlear cell clusters. The top 5 deferentially expressed (DE) genes for the 26 identified clusters are shown. Cellular identity for each cluster is indicated by a color bar at the top of the heat map. The color ranges from blue to bright yellow indicates low to high gene expression levels, respectively.

The cochlear duct cells were isolated from the P1 and P7 cochlear duct and applied to scRNA-seq analysis (Figure 1A). The results obtained from Cell Ranger analyses were shown in Supplementary Figure 1. The estimated number of cells in the current study was 28557. Fraction reads in cells were 91.1%. Mean reads per cell were 38929 in P1 and 32218 in P7. Median genes per cell were 2151 in P1 and 2283 in P7. Total genes detected were 20866 in P1 and 20367 in P7. Reads Mapped to Genome were 96.20% in P1 and 96.30% in P7 (Supplementary Data 1). Following the quality control of scRNA-seq data, we retained 12826 cells in P1 and 12313 cells in P7 for the downstream analysis (Supplementary Data 2). After aggregated and normalized scRNA-seq data, t-distributed stochastic neighbor embedding (t-SNE) analysis (Satija et al., 2015) using Seurat R Package was performed for cell-type identification (Butler et al., 2018). The results of single-cell sequencing analysis showed that a total of 26 cell clusters were identified in P1 and P7 phases. An examination of the top five genes defining each cluster was used to assign identities to each group. Figure 1B showed the cell clusters in the P1 phase, and Figure 1C showed the cell clusters in the P7 phase. Visualization of the top five most variably expressed genes between cell clusters showed distinct transcriptional programs of the 26 clusters (Figure 1D).

The expression patterns of enriched genes of each cluster were analyzed. Figure 2 shows the high expressed gene in each cell group. The darker the color, the richer the expression level of these genes in this cell’s cluster. Cldn5, Loc100910270, Mt3, Plvap, Epcam, Stmn2, Rgs5, Lyz2, Ube2c, Gjb6, Gcg, Slco1a4, Cenpf, Coch, Aldh1a2 genes are highly expressed on one of the clusters, while Crym, Gpc3, Ccn3, Dbi, Col9a1, Sptssa, Pmp22 genes are co-express on several clusters (Figure 2). t-SEN plots of most ten abundant genes of each cell cluster were shown in Supplementary Figures 2–28).

**FIGURE 2:**
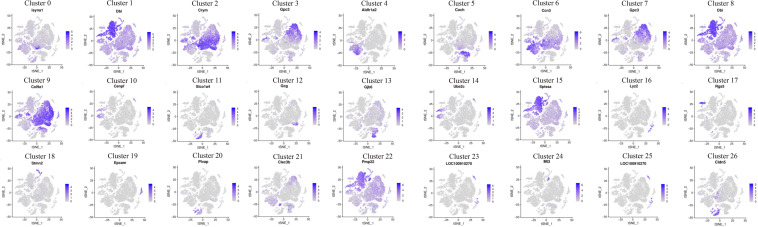
Cochlea cells landscape revealed by scRNA-seq analysis. scRNA-seq was performed on single-cell suspensions pooled from P1 and P7 cochlea duct. All samples were analyzed using canonical correlation analysis with the Seurat R package. Cells were clustered using a graph-based shared nearest-neighbor clustering approach plotted by tSNE plot. Feature Plots of the most abundant gene in each cell cluster of cochlea cells.

### scRNA-Seq Identifies Four GER Cell Clusters According to the Cells Number Dynamic Change From P1 to P7, and Gene Expression for Significant Marker Genes

**FIGURE 3:**
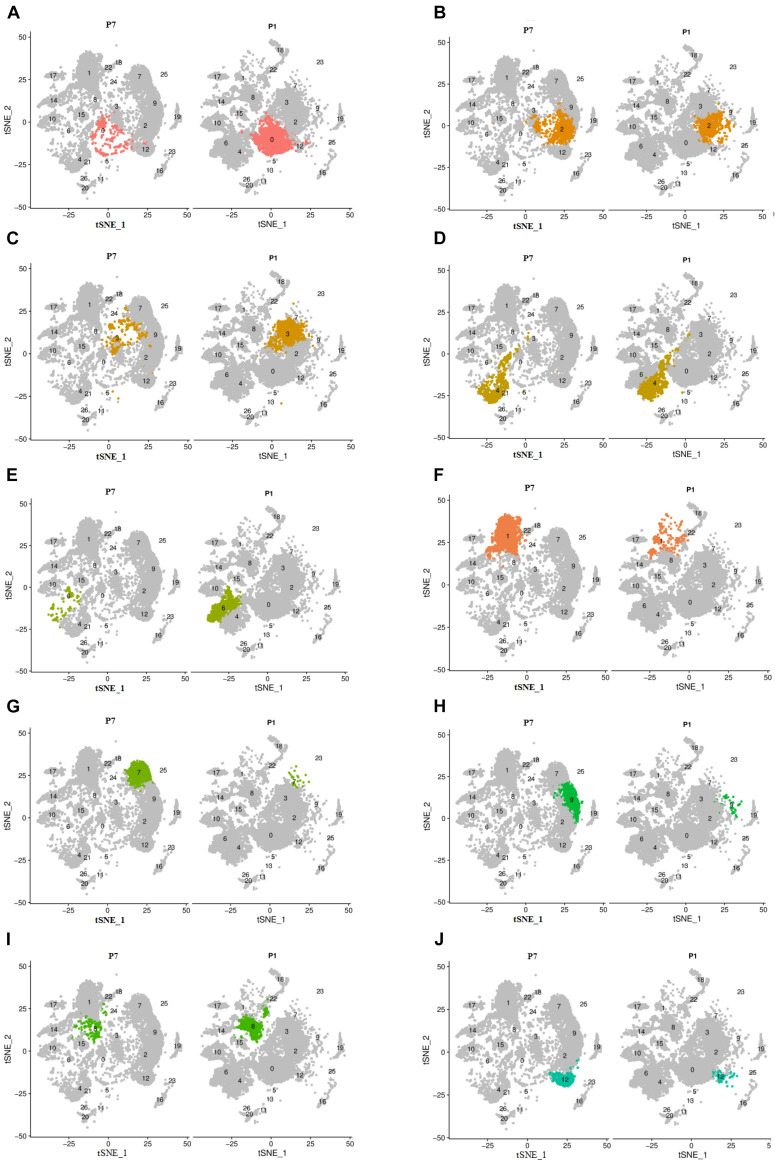
The change of cell number of the above different cell clusters in two different periods of P1 and P7. Cells were clustered using a graph-based shared nearest-neighbor clustering approach plotted by a tSNE plot. **(A,C–E,I)** The decreased clusters of 0, 3, 4, 6, and 8. **(B,F–H,J)** The significantly increased clusters of 1, 7, 9, and 12.

Analysis of the cell numbers of each cell cluster of P1 and P7 showed that there was a significant decrease in the cell number of clusters 0, 2, 3, 4, 6, and 8, with statistically significant differences in clusters 0, 3, 6, and 8. And the cell number of clusters 1, 7, 9, and 12 increased and had statistical differences, as shown in Supplementary Data 2 and Figure 3.

**FIGURE 4:**
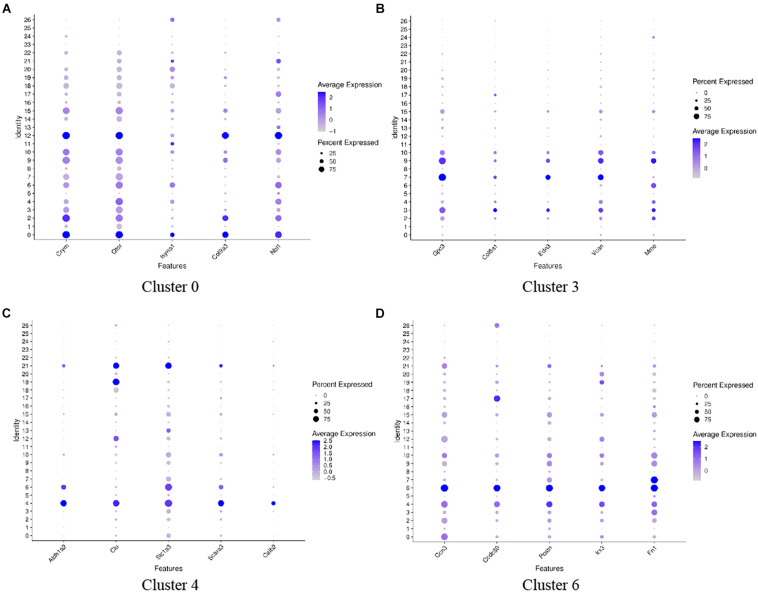
Dot plots representing expression levels of cochlea cells. scRNA-seq was performed on single-cell suspensions pooled from the cochlea duct of SD rats including P1 and P7 stages. All samples were analyzed using Dot plot analysis with the Seurat R package. Expression levels of the top five genes on cluster 0 **(A)**, cluster 3 **(B)**, cluster 4 **(C)**, cluster 6 **(D)** are shown. Each dot was sized to represent the proportion of cells of each type expressing the marker gene and colored to represent the mean expression of each marker gene across all cells, as shown in the key.

Great epithelial ridge cells are transient structures during cochlear development (Dayaratne et al., 2014), and a typical manifestation of this is a gradual decrease in the number of cells during auditory development. We performed genotypic analysis on these five cell clusters with significantly reduced cell numbers: clusters 0, 3, 4, 6, and 8. Figure 4 shows the top five genes with high expression in each cell cluster (Figure 4). Oto and Crym were relatively highly expressed on cluster 0 (Figure 4A), and these two genes were also highly expressed on cluster 2 and cluster 12. On the t-SNE plot, cluster 12 and cluster 0 were closely linked in spatial position. Vcan, Edn3, and Gpc3 had similar gene expression patterns in clusters 3, 7, and 9 (Figure 4B). Scara3 and Aldh1a2 were relatively specifically highly expressed on cluster 4 (Figure 4C). More specific marker genes were present on cluster 6, including Ccn3, Irx3, Scg3, and Postn. Slc1a3 and Postn were similarly expressed on clusters 4 and 6 (Figure 4D). Cluster 8 lacked significant specific highly expressed genes and differed significantly from cluster 0, 3, 4, and 6 in gene expression patterns and spatial location.

**FIGURE 5:**
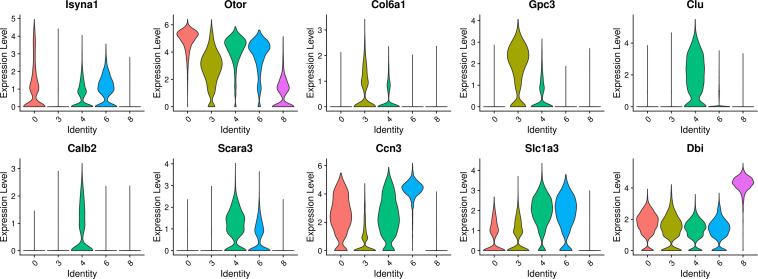
Violin plots showing select genes that are deferentially expressed in the decreased clusters of 0, 3, 4, 6, and 8. *Y*-axis, log-normalized transcript counts.

The select genes with high expression in the decreased clusters of 0, 3, 4, 6, and 8 were further analyzed and the results are shown in Figure 5. In terms of gene expression, clusters 0, 3, 4, and 6 have similar gene expression patterns. Separately, Isyna1 was more highly expressed on cluster 0; Col6a1 and Gpc3 were more highly expressed on cluster 3; Otor and Ccn3 had similar high expression levels in cluster 0, 3, 4, and 6; Clu, Scara3, and Calb2 were significantly and specifically highly expressed on cluster 4; Slc1a3 had similar expression levels on cluster 4 and 6. In contrast to cluster 8, most of these aforementioned genes showed a lower expression on cluster 8, but the gene of Dbi was relatively highly expressed on cluster 8, suggesting that cluster 8 and the other four cell clusters are two cell types of different nature.

### Gene Ontology (GO) Function Analysis Showed These Four Identified GER Cell Clusters Have Similar Expression Patterns

**FIGURE 6:**
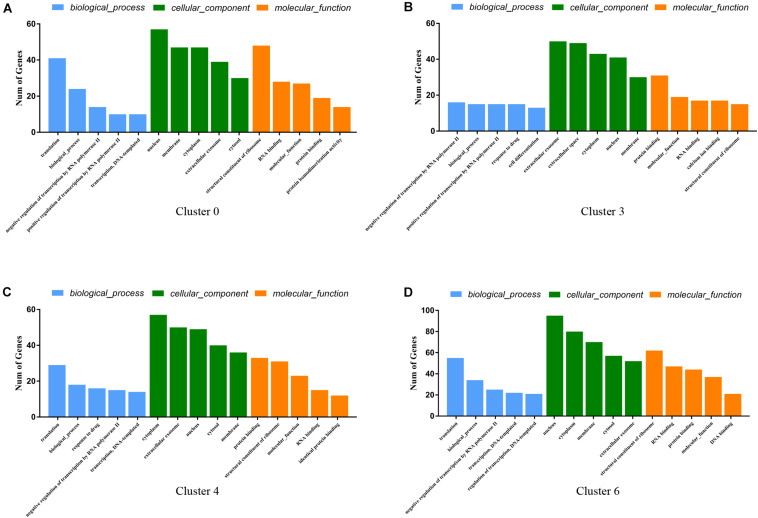
GO enrichment analysis of genes for cluster 0 **(A)**, cluster 3 **(B)**, cluster 4 **(C)**, cluster 6 **(D)**. The Go functions include biological processes (BP, blue color), cellular components (CC, green color), and molecular functions (MF, yellow color). The horizontal coordinate-axis *X* is the item of go function, and the ordinate represents the genes enriched by different items.

Gene Ontology function consists of biological processes (BP), cellular components (CC), and molecular functions (MF). We analyzed the GO functions of clusters 0, 3, 4, 6 and found the following characteristics. The gene of cluster 0 is mainly enriched in Translocation (BP), Nucleus (CC), and Structural constituent of Ribosome (MF) (Figure 6A). The gene of cluster 3 is mainly enriched in Negative regulation of transcription from RNA polymerase II, Biological process, Positive regulation of transcription from RNA polymerase II, Cell differentiation and translation (BP), Extracellular exosome, Extracellular space, Cytoplasm and Nucleoplasm, Cytoplasm, and Nucleus (CC) and Protein binding (MF) (Figure 6B). The gene of cluster 4 has similar BP and CC as cluster 3 that are mainly enriched in Translation, Cytoplasm, Extracellular exosome, and Nucleus (Figure 6C). Besides, cluster 4 is similar to cluster 0 in terms of MF and is enriched in Protein binding and Structural constituents of the Ribosome. Cluster 6 is similar to cluster 2 in terms of BP, mainly enriched in Translocation, Biological process, negative regulation of transcription from RNA polymerase II (Figure 6D). Also, in the CC, cluster 6 is mainly enriched in Cytoplasm, Nucleus, and Membrane, similar to cluster 3 and cluster 4 (Figures 6B,C), while enriched in Structural constituent of Ribosome, RNA-binding, and Protein binding which is similar to cluster 0 in MF, which shows that these cell clusters have similar expression patterns.

### KEGG Pathway Analysis for the Identified GER Cell Clusters

**FIGURE 7:**
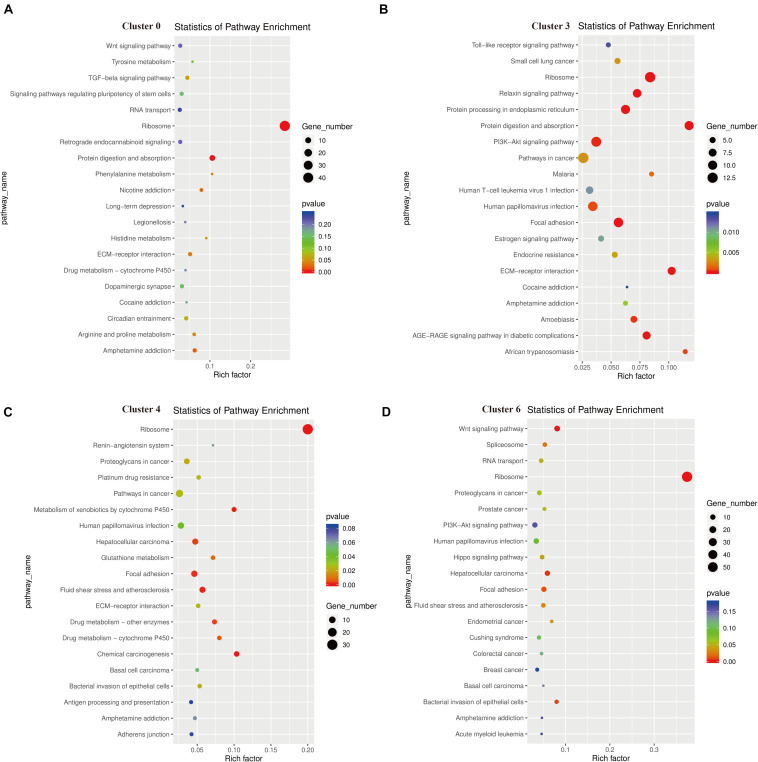
Functional enrichment analyses using Kyoto Encyclopedia of Genes and Genomes (KEGG) pathways for cluster 0 **(A)**, cluster 3 **(B)**, cluster 4 **(C)**, cluster 6 **(D)**. The triangle size indicates the significance and corresponding significance values displayed as log10 (*P*-value).

The results of KEGG signaling pathway analysis for cluster 0, cluster 3, cluster 4, and cluster 6 are shown in Figure 7. The results showed that genes in all clusters were predominantly enriched in Ribosome signaling pathway (Figures 7A–D). In cluster 0, 44 genes are rich in Ribosome, and the top 5 genes are Rps16, Rps18l1, Rpsa, Rps5, Rps17 (Supplementary Data 3). In cluster 3, there were 13 genes enriched in Ribosome, and the top 5 genes are Rps16, Rps8, Rpl41, Rpl28, Rps12 (Supplementary Data 4). In cluster 4, there were 31 genes enriched in Ribosome, and the top 5 genes are Rps18l1, Rps5, Rps17, Rpl10a, Rps8 (Supplementary Data 5).

In cluster 6, there were 58 genes enriched in Ribosome, and the top 5 genes are Rps16, Rps19, Rps18l1, Rpsa, Rpl35 (Supplementary Data 6). We found that Rp16 was involved in the Ribosome signaling pathway of these three cell clusters. While in cluster 3, some genes were also clearly enriched in the P13K-Akt signal pathway, Focal adhesion, and Protein digestion and absorption. The genes enriched in the P13K-Akt signal pathway are Col4a2, Col6a2, Creb3l1, AABR07068316.1, Igf2, Col4a1, Gf1, Col6a1, Fn1, Pten, Col1a1, Spp1, and genes enriched in Protein digestion and absorption are Col5a2, Col4a2, Col6a2, Col3a1, Mme, AABR07068316.1, Col5a1, Col4a1, Col6a1, Col1a1 (Supplementary Data 4). In addition, we found the main genes in the apoptotic pathway were Ctsk, Fos, Ctsb, and Jun.

### The Heterogeneity Analysis of These Four GER Cell Clusters

**FIGURE 8:**
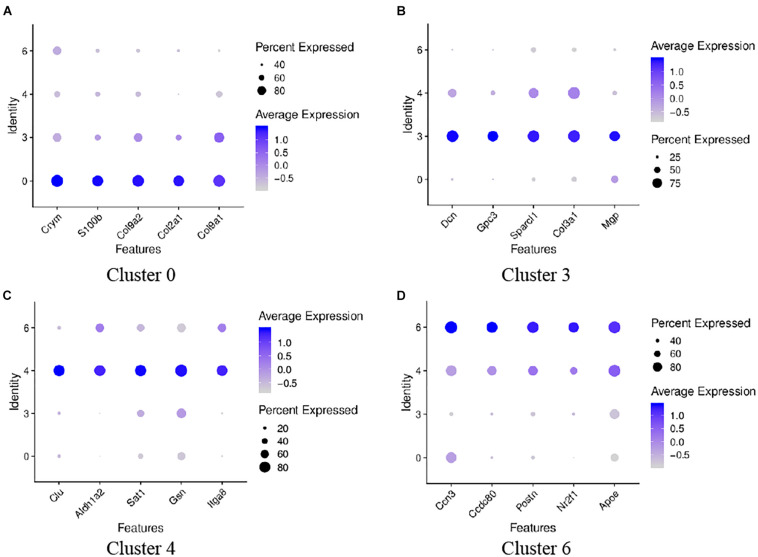
Dot plots representing expression levels of the four GER cells. A re-clustered analysis was performed to reveal the heterogeneity of differential genes expression. Expression levels of the top five genes on cluster 0 **(A)**, cluster 3 **(B)**, cluster 4 **(C)**, cluster 6 **(D)** are shown. Each dot was sized to represent the proportion of cells of each type expressing the marker gene and colored to represent the mean expression of each marker gene across all cells, as shown in the key.

To analyze the heterogeneity of these four cell clusters, we performed a re-clustered analysis. Analysis of the top ten genes with high expression showed that the top five genes expressed on cluster 0 were Col9a1, Col2a1, Crym, Col9a2, and S100b, respectively; the top five genes expressed on cluster 3 were Dcn, Sparcl1, Col3a1, Gpc3, and Mgp, respectively. The top five expressed genes on cluster 4 were Gsn, Clu, Sat1, Itga8, and Cavin2. The top five expressed genes on cluster 6 were Apoe, Ccn3, Postn, Ccdc80, and Nr2f1. Figure 8 shows the top five genes highly expressed in the above four cell clusters.

**FIGURE 9:**
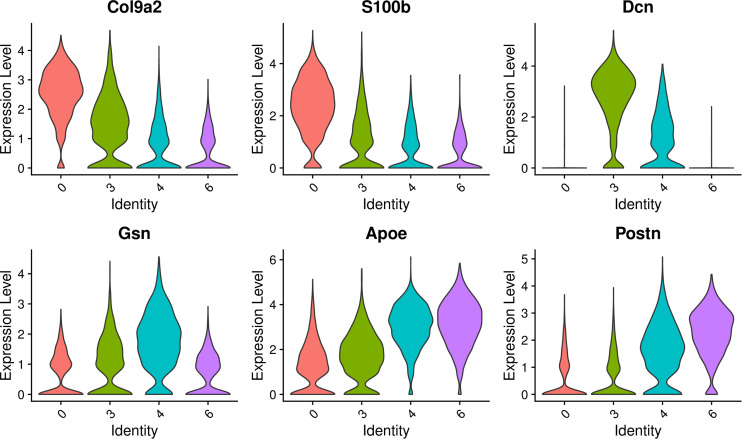
Expression of select genes of these four GER cell clusters. Violin plots showing normalized log-transformed expression values for the select genes for cluster 0, cluster 3, cluster 4, cluster 6.

The select genes of these four cell clusters were analyzed to reveal the heterogeneity, and the results are shown in Figure 9. In terms of gene expression, Col9a2 and S100b was more highly expressed on cluster 0 and 3; Dcn was more highly expressed on cluster 3; Gsn had similar high expression levels in cluster 3 and 4; Postn was highly expressed on cluster 4 and 6; Apoe had similar expression levels on this four clusters. These findings indicate that these four GER cell clusters are some heterogeneity.

**FIGURE 10:**
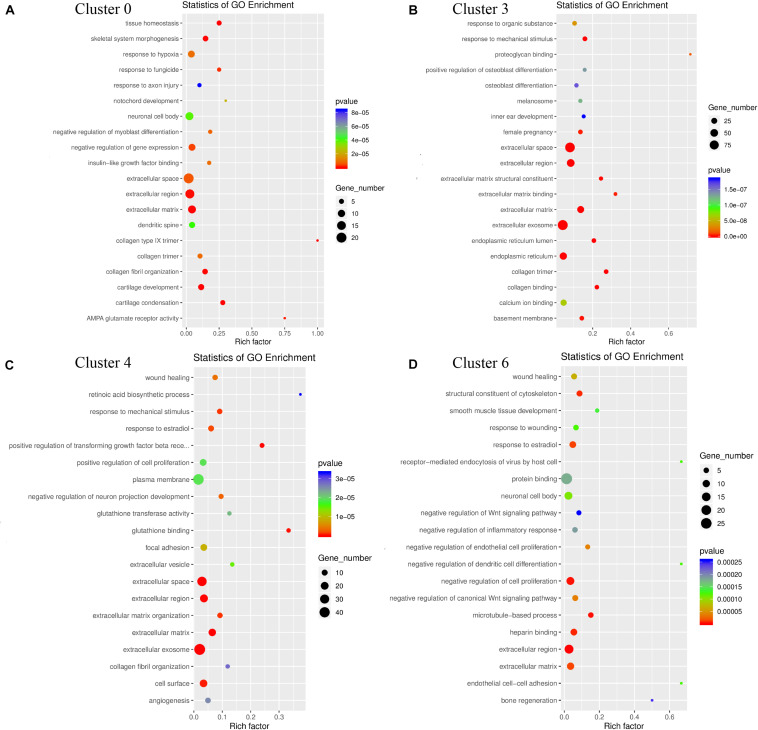
GO enrichment analysis of genes for the heterogeneity of cluster 0 **(A)**, cluster 3 **(B)**, cluster 4 **(C)**, cluster 6 **(D)**. The triangle size indicates the significance and corresponding significance values displayed as log10 (*P*-value).

The results of GO functional enrichment analysis showed that the genes in cluster 0 were mainly enriched in the extracellular space and extracellular region; the genes in cluster 3 and cluster 4 were mainly enriched in the extracellular exosome and extracellular space; the genes in cluster 6 are mainly enriched in the extracellular region and negative regulation of cell promotion. These results showed that they had similar GO functions, and the genes were mainly enriched in regulating the changes of cell spatial structure, among which cluster 3 and cluster 4 had higher homogeneity. Cluster 6 plays a major role in the negative regulation of cell proliferation (Figure 10).

**FIGURE 11:**
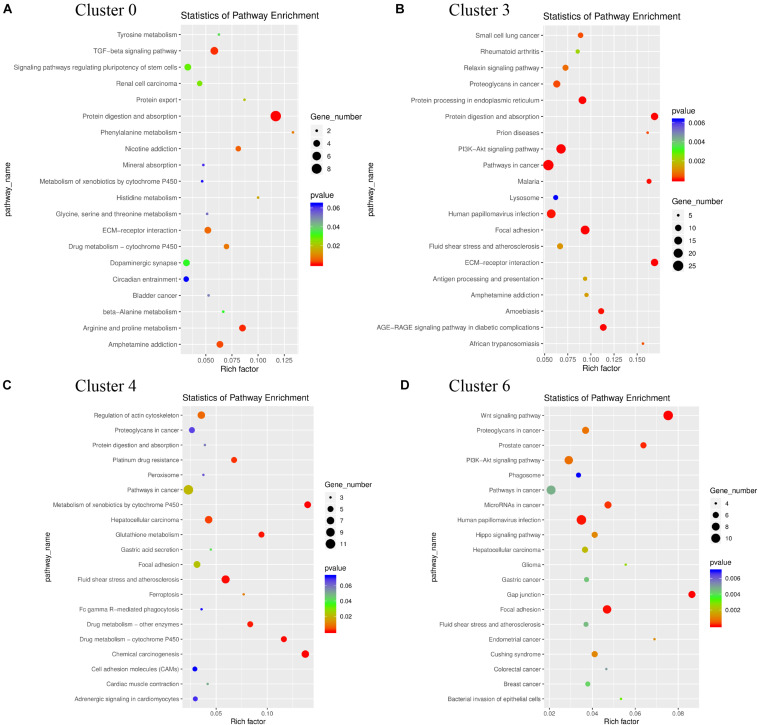
KEGG pathways functional enrichment analyses for the heterogeneity of cluster 0 **(A)**, cluster 3 **(B)**, cluster 4 **(C)**, cluster 6 **(D)** after re-clustered. The triangle size indicates the significance and corresponding significance values displayed as log10 (*P*-value).

KEGG signaling pathway analysis showed that the genes in cluster 0 were mainly enriched in protein digestion and absorption and TGF beta signaling pathway, which regulated the digestion and absorption of cell debris after apoptosis; The genes in cluster 3 are mainly enriched in the PI3K-Akt signaling pathway and pathways in cancer, which regulates cell apoptosis; The genes in Cluster 4 are mainly enriched in fluid shear stress and atherosclerosis, and hepatocellular carcinoma, which also plays a role in regulating apoptosis. The genes in cluster 6 were mainly enriched in Wnt signaling pathway, and human papillomavirus infection (Figure 11). According to these results, the four-cell clusters are heterogeneous.

### Fluorescence *in situ* Hybridization (FISH) of GER Cell Clusters

**FIGURE 12:**
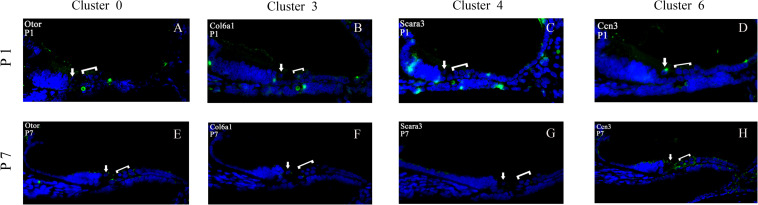
Validation of high expression gene of GER cell clusters at P1 and P7. Otor was high and nearly restricted expressed to the whole GER cells at P1 (green color), and down-regulated expression in GER at P7 **(A,E)**. Col6a1 was significantly expressed in EGR region of the cochlea at P1, but almost disappeared at P7 **(B,F)**. Scara3 was centrally expressed in the lateral wall of the GER region of the cochlea during the P1 period, but showed a significantly reduced scattered expression during the P7 period **(C,G)**. The location of Ccn3 expression in the GER region of the cochlea during P1 overlapped a lot with Otor, but the expression was also significantly reduced at P7 **(D,H)**. For all panels, the IHC is indicated with an arrow and the three OHCs are indicated by a bracket.

To validate the cell-type-specific genes of clusters 0, 3, 4, and 6, we used fluorescence *in situ* hybridization (FISH) to localize transcripts for these four GER cells types in cross-sections from P1 to P7 cochlea (Figure 12). Four genes with the high expression on clusters 0, 3, 4, and 6 based on scRNA-seq results were selected for FISH: Otor, Col6a1, Scara3, and Ccn3. All four genes showed patterns of expression that were consistent with the single-cell results. Otor that was detected in all GER cell clusters, and was among the top five differentially expressed genes in cluster 0. From the FISH result, it can be seen that Otor was high and nearly restricted expressed to the whole GER cells at P1 (green color), and down-regulated expression in GER at P7 (Figures 12A,E). Col6a1 was significantly expressed in EGR region of the cochlea at P1, but almost disappeared at P7 (Figures 12B,F). Scara3 was centrally expressed in the lateral wall of the GER region of the cochlea during the P1 period but showed a significantly reduced scattered expression during the P7 period (Figures 12C,G). The location of Ccn3 expression in the GER region of the cochlea during P1 overlapped a lot with Otor, but the expression was also significantly reduced at P7 (Figures 12D,H).

## Discussion

As a temporary structure in the development of the cochlea, the presence of GER indicates that the cochlea is still immature (Bryant et al., 2002; Inoshita et al., 2014; Peeters et al., 2015; Mazzarda et al., 2020). GER cells undergo cellular morphological changes after birth, such as plasma membrane separation, cell shrinkage, cell gap enlargement, and columnar cells replaced by cubic cells (Uziel, 1986). At the same time, the number of cells is greatly reduced and eventually changes to a mature inner sulcus region (Hinojosa, 1977; Woods et al., 2004; Sirko et al., 2019). The present single-cell RNA sequencing results confirmed that the GER cell population decreases over time, in accord with prior work (Hinojosa, 1977; Woods et al., 2004; Sirko et al., 2019). The GER has an important role in the survival and maturation of auditory neurons, synaptic development, and refinement of auditory afferent and efferent innervation before the emergence of hearing (Chai et al., 2012; Johnson et al., 2017; Mammano and Bortolozzi, 2018; Ceriani et al., 2019). There is a high degree of morphological uniformity in a range of cell shapes in the GER region, but it is not clear whether different subtypes of cells play different regulatory roles during cochlea development (Dayaratne et al., 2014; Hayashi Y. et al., 2020). Kolla et al. (2020) classified GER cells into four different subtypes by single-cell sequencing analysis, and genes with different expression abundance on different cell subtypes. The authors named the cells as L.KO, lateral Kölliker’s organ cells and M.KO, medial Kölliker’s organ cells according to their expression patterns, and further divided the L.KO cells into KO1, KO2, and KO3 subtypes. Among them, KO1 cell cluster highly expresses Dcn and Rcn3; KO2 cell cluster highly expresses Cpxm2, Ctgf, Kazald1, and Tectb; KO3 cell cluster highly expresses, Gjb6, Net1, Tectb, and Tsen15; KO4 cell cluster highly expresses Calb1, Clu, Crabp1, Epyc, and Itm2a. Kubota et al. (2021) classified GER cells into S2, S3, and S4 cell subtypes based on specific gene expression. Among them, Gsn and Sparcl1 were significantly highly expressed on all three cell subtypes, Crabp1 was significantly highly expressed on the S2 cell subtype, and Scara3, Clu, and Gpc3 were highly expressed on the S2 and S3 cell subtype. From these results, three distinct GER cells groups that correlate with a specific spatial distribution of marker genes were identified, and disappeared during post-natal cochlear maturation.

In the present study, we compared cell cluster typing and numbers at P1 and P7. The results showed that the cells of clusters 0, 3, 4, 6, and 8 were significantly reduced over time. Clusters 0, 3, 4, and 6 showed high similarity in the expression patterns, GO functions, and signaling pathways. Besides, these four clusters were also closely related to each other on the tSNE plot, while cluster 8 had no spatial relationship with these clusters. Based on these results, we considered that clusters 0, 3, 4, and 6 were different subtypes of GER cells.

At the same time, we found that clusters 2, 7, 9, and 12 were highly similar to clusters 0 and 3 in terms of gene expression patterns, and the cell numbers of these four clusters showed an increase around P7, with a statistically significant difference in the cluster 7, 9, and 12. Vcan, Edn3 and Gpc3 had similar gene expression patterns in clusters 3, 7, and 9. Oto and Crym were closely linked in spatial locations between cluster12 and 0, and their spatial locations were close to each other. The research of Kubota et al. (2021) is consistent with this study. Kubota et al. (2021) believe that the most lateral GER group has the highest similarity with neonatal inner border and inner phalangeal cells, and thought these inner border and inner phalangeal cells have a similar organ-forming potential. However, whether these cell populations can also be considered as different subtypes of GER cells or other types of cochlear support cells that disappear after full maturation of cochlear hearing development still requires further in-depth investigation.

Although, the number of cells in clusters 0, 3, 4, and 6 decreased significantly from P1 to P7, the detailed mechanism is unclear.

Yang and He (2016) found that the morphology of GER cells in the newborn rat cochlea gradually appeared to replace the short columnar epithelium with high columnar cells from the basilar turn to the apex as they developed, and the number of cells also gradually decreased. They hypothesized that GER cells apoptosis played an important role in the development of rat cochlea. In addition, GER cells exhibited programmed apoptosis from the basilar turn to the apex turn *in vivo* experiments, but showed proliferation *in vitro* experiments (He and Yang, 2015b). The authors suggested that the initiating factors of apoptosis might come from outside of the GER cells rather than from intrinsic cellular factors. It was also found (Hou et al., 2019, 2020) that the expression levels of caspase-3, caspase-8, caspase-9, and Bcl-2 gene mRNA and protein in the basilar membrane of rat cochlea at different times after birth were significantly time-dependent. Together, those studies suggest that some GER cells undergo apoptosis while other proliferate. However, proliferating cells are outnumbered by apoptotic cells, which eventually leads to the disappearance of GER cells. Autophagy is also thought to be involved in GER cells development and both autophagy and apoptosis show a strict time dependence, with peak activity occurring at P1 or earlier in autophagic, and apoptosis occurring at P7 or later (He and Yang, 2015b; Yang and He, 2016). Autophagy and apoptosis play different roles in different stages of cochlea development (Takahashi et al., 2001; Peeters et al., 2015; Liu et al., 2017; Hayashi K. et al., 2020). Disruption of autophagy or apoptosis of supporting cells during cochlea development will result in impaired development or hearing impairment (He et al., 2017; Mammano and Bortolozzi, 2018; Zhou et al., 2020). Therefore, the dynamic balance between autophagy and apoptosis regulates the normal differentiation and development of the cochlea, but the specific regulatory mechanism is not yet clear.

The results of this scRNA sequencing showed that GER clusters have many commonalities in Go function enrichment. In terms of biological processes, the enrichment is more consistent in Translation and Negative regulation of transcription from RNA polymerase II; in terms of cellular components, the gene enrichment is more consistent in Nucleus and Cytoplasm; in terms of molecular functions, the enrichment is mainly in Protein binding and Structural constituent of Ribosome. In terms of molecular mechanisms, transcription and translation are very active. Meanwhile, different GER cell clusters, such as clusters 3 and 4, have a large number of genes enriched in Negative regulation of transcription from RNA polymerase II, which play a negative dynamic regulatory role (Sun et al., 2015). These negatively regulated genes are mainly Nedd4, Rarb, Foxp2, Pawr, Dact1, Igf2, Egr1, H2afy2, Btg2, Calr, Foxc1, Mdfi, Rps14, Jun, Peg3, and Ets2.

At present, several genes and signaling pathways have been confirmed to play important roles in the development of the inner ear, such as the Sox2 (Yang et al., 2019), Pax2 (Patel et al., 2018), Atoh1 (Zhong et al., 2019), FGF (Yang et al., 2018) Notch (Daudet and Żak, 2020, FoxG1 (Ding et al., 2020), Shh (Bok et al., 2007), mTOR (Fu et al., 2018), and Wnt (Waqas et al., 2016) pathways. The results based on the KEGG signaling pathway showed a high degree of consistency in gene enrichment in these four GER cell clusters. Clusters 0, 4, and 6 were significantly enriched in the Ribosome signaling pathway (see Figures 7A,C,D); cluster 3 was enriched in PI3K-Akt and Protein digestion and absorption signaling pathway in addition to the Ribosome signaling pathway (see Figure 7B), which is consistent with the Go function results. The ribosome signaling pathway is an important signaling pathway regulating development, and ribosome biosynthesis is one of the most multifaceted and energetically demanding processes in the whole of biology, involving protein assembly and maturation factors, and requiring the coordinated involvement of multiple cellular functions (Pelletier et al., 2018). Mitosis is a key process of organ development and maturation, and vigorous mitosis suggests cells are dividing and proliferating, yet the overall number of GER cells decrease during postnatal development, presumably mainly related to the negative regulatory signaling pathway of cluster 3. The PI3K-Akt signaling pathway is an important signaling pathway that regulates cell proliferation, differentiation, apoptosis, and migration (Ediriweera et al., 2019; Jia et al., 2019). It has also been shown to regulate hair cell regeneration in cochlea developmental regeneration studies (Mullen et al., 2012; Xia et al., 2019).

Cluster 3 has a large number of genes enriched in the PI3K-Akt signaling pathway, including Col4a2, Col6a2, Creb3l1, AABR07068316.1, Igf2, Col4a1, gf1, Col6a1, Fn1, Pten, Col1a1, Spp1, which might regulate the proliferation and apoptosis of GER cells, and through Col5a2, Col4a2, Col6a2, Col3a1, Mme, AABR07068316.1, Col5a1, Col4a1, Col6a1, Col1a1 on the Protein digestion and absorption signaling pathway regulate these apoptotic proteins and autophagy of cellular debris, thereby directing the orderly degeneration of GER cells or possible trans-differentiation of hair cells or supporting cells. In this study, we also found that the genes enriched in the apoptotic pathway are mainly Ctsk, Fos, Ctsb, and Jun. The mechanism of these genes in regulating the degeneration of GER cells and promoting auditory development has not been reported, and the role of this signaling pathway and related genes still needs to be investigated in depth.

## Limitation of Study

The present study has the following shortcomings: (1) We did not perform localization studies of the characteristic genes expressed in these four GER subtypes; (2) Previous morphological studies showed that GER cells in rodents degenerate and disappear in 12–14 days after birth, by which time auditory function emerges. In the present study, we only studied GER cells subtypes up to P7, and found a significant decrease in the number of GER cells, but not a complete degeneration; (3) We also found that clusters 2, 7, 9, and 12 have an increased number of cells and their gene expression patterns are common to the above four subtypes of GER cells. Whether these cells are trans-differentiated hair cells, supporting cell precursor cells, or other subtypes of GER cells, and the fate transition of these cells still needs more in-depth study and exploration; (4) Although we identified several gene candidates, we did not perform mechanistic studies to determine the relationship of these genes and related signaling pathway with proliferation, apoptosis, and autophagy of GER cells.

## Data Availability Statement

The original raw data had been upload on the GEO database. The GEO accession number is GSE170810, and view at: https://www.ncbi.nlm.nih.gov/geo/query/acc.cgi?acc=GSE170810.

## Ethics Statement

The animal study was reviewed and approved by Animal Ethics Committee of Shanghai Jiao Tong University School of Medicine.

## Author Contributions

JY, FM, and GZ: study design. JiC, DG, JuC, SH, BH, and LS: acquisition of data. YL, SL, FZ, and XS: analysis and interpretation of data. JiC and DG: drafting the manuscript. JY and GZ: study supervision. All authors contributed to the article and approved the submitted version.

## Conflict of Interest

The authors declare that the research was conducted in the absence of any commercial or financial relationships that could be construed as a potential conflict of interest.

## Publisher’s Note

All claims expressed in this article are solely those of the authors and do not necessarily represent those of their affiliated organizations, or those of the publisher, the editors and the reviewers. Any product that may be evaluated in this article, or claim that may be made by its manufacturer, is not guaranteed or endorsed by the publisher.

## Footnotes

1
https://support.10xgenomics.com/single-cell-gene-expression/software/downloads/3.1/
