# Supplementary figures and images for: Single-Cell RNA Sequencing Analysis Reveals Greater Epithelial Ridge Cells Degeneration During Postnatal Development of Cochlea in Rats
Source: Front Cell Dev Biol. 2021 Sep 3;9:719491. doi: 10.3389/fcell.2021.719491 (PMC8446670; doi:10.3389/fcell.2021.719491)

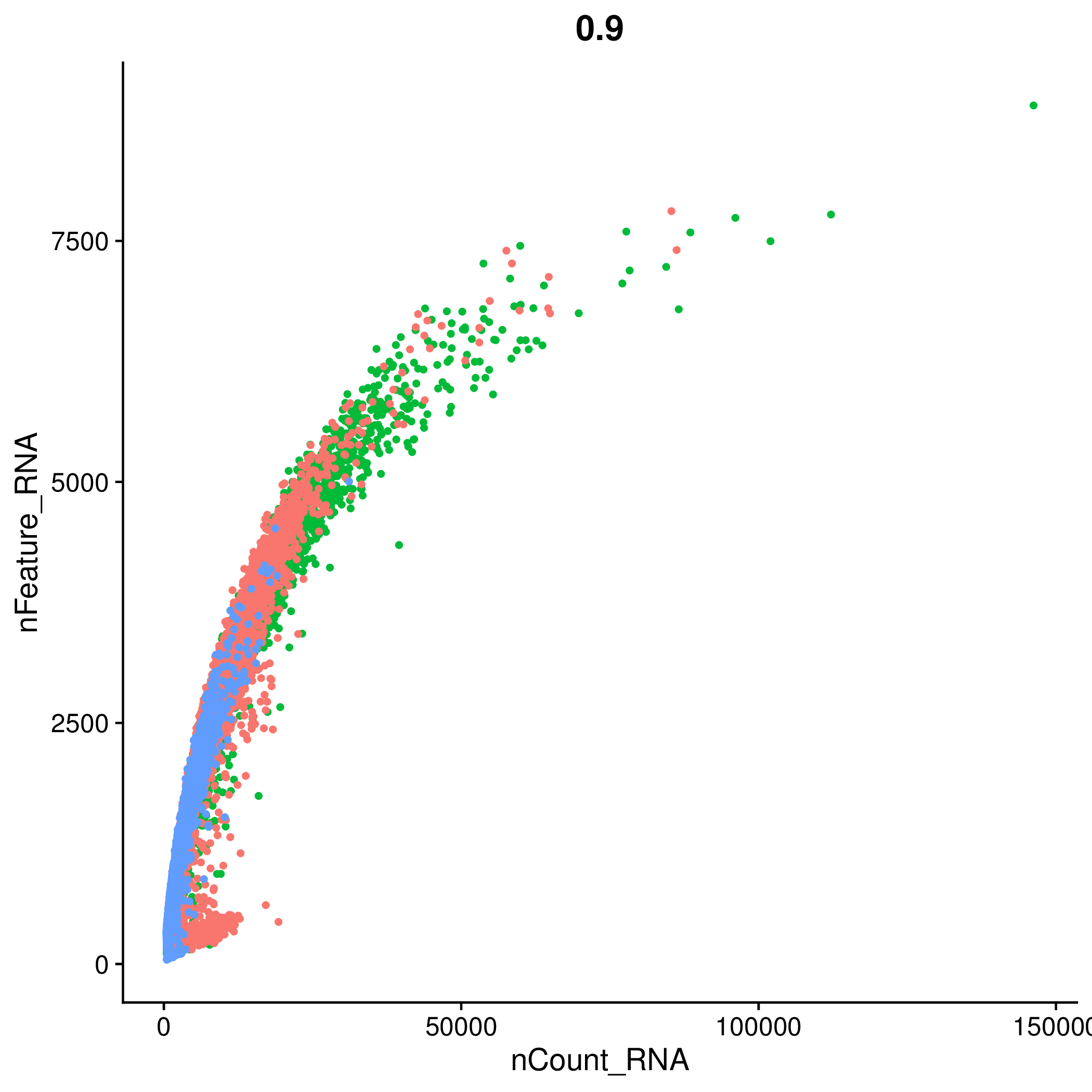

Supplement: Supplementary file 1 [file Data_Sheet_1.ZIP › S1.png]

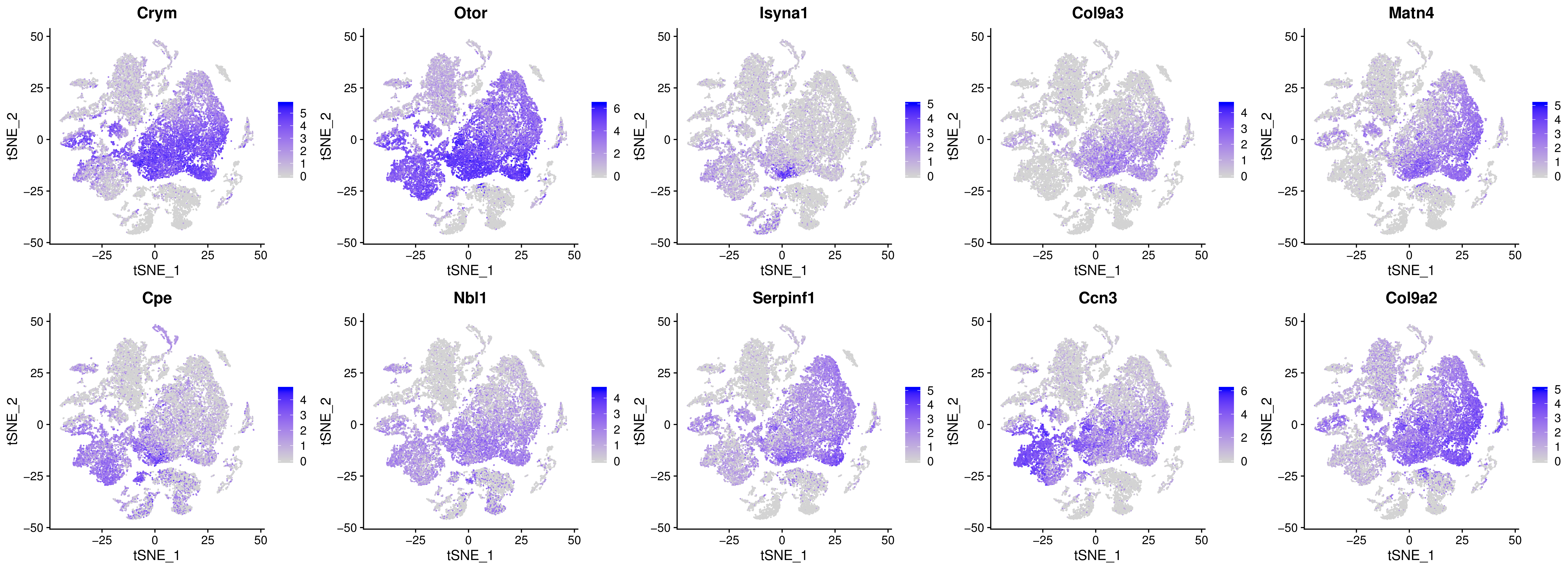

Supplement: Supplementary file 1 [file Data_Sheet_1.ZIP › S2.png]

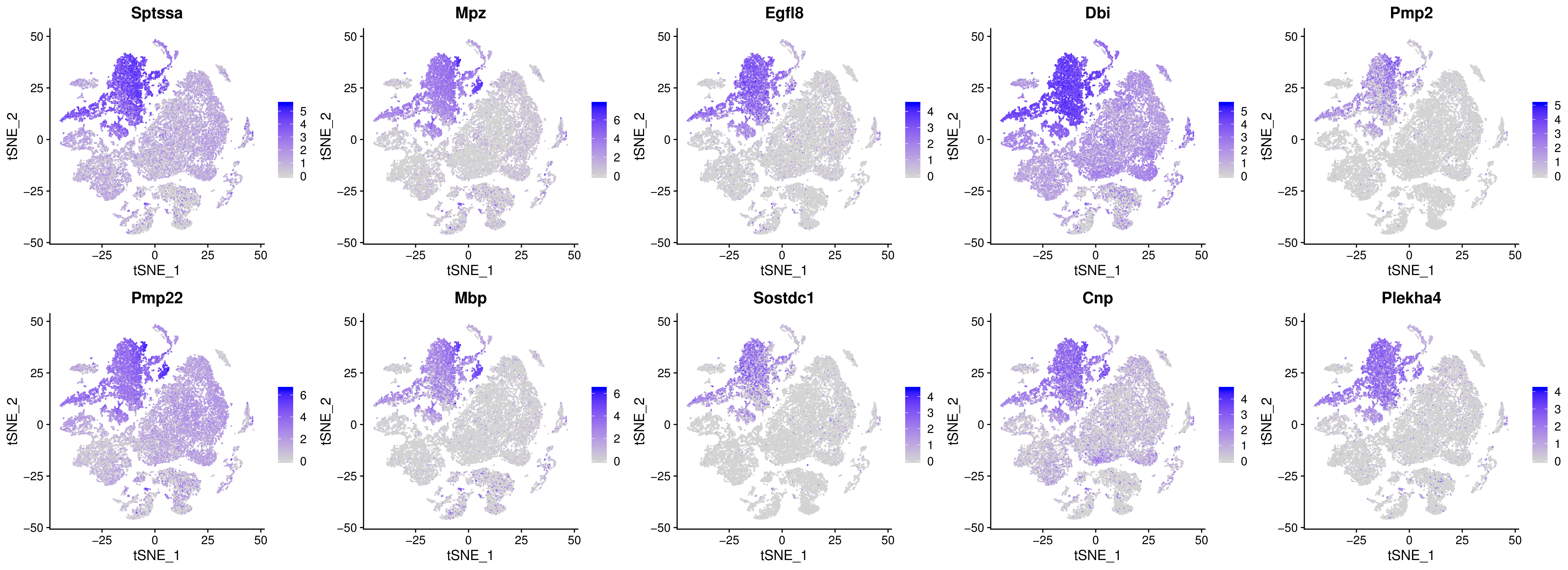

Supplement: Supplementary file 1 [file Data_Sheet_1.ZIP › S3.png]

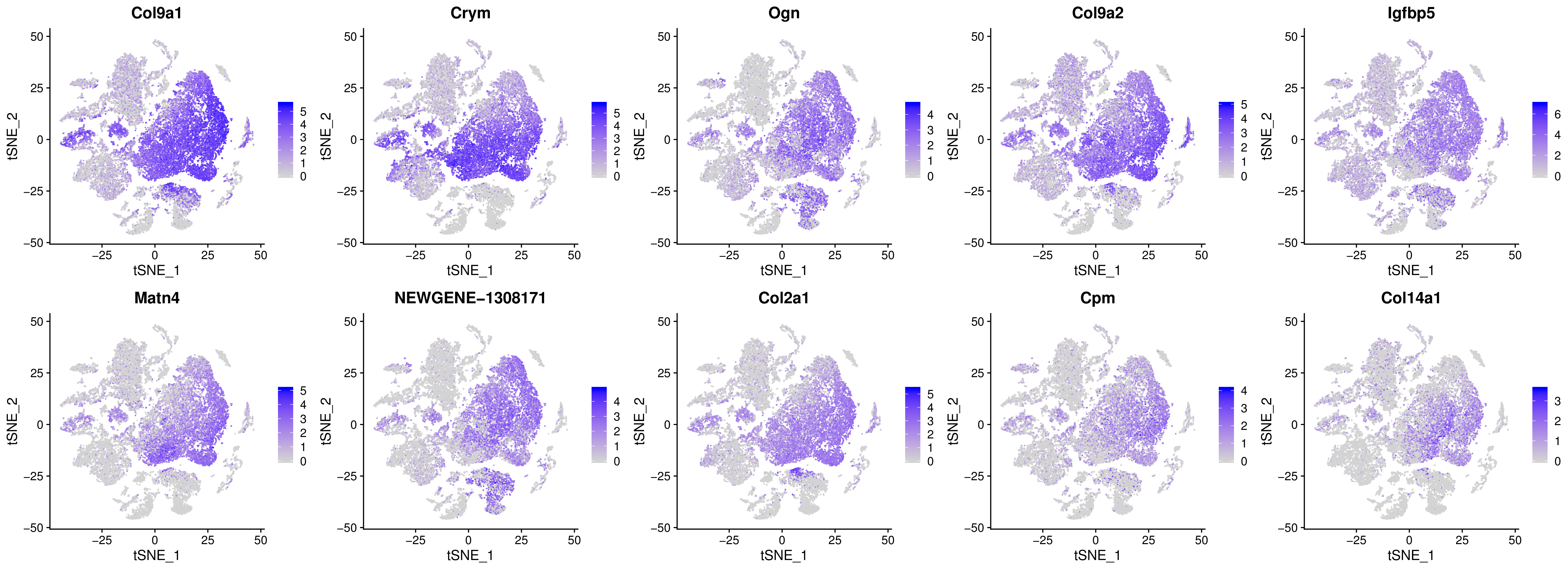

Supplement: Supplementary file 1 [file Data_Sheet_1.ZIP › S4.png]

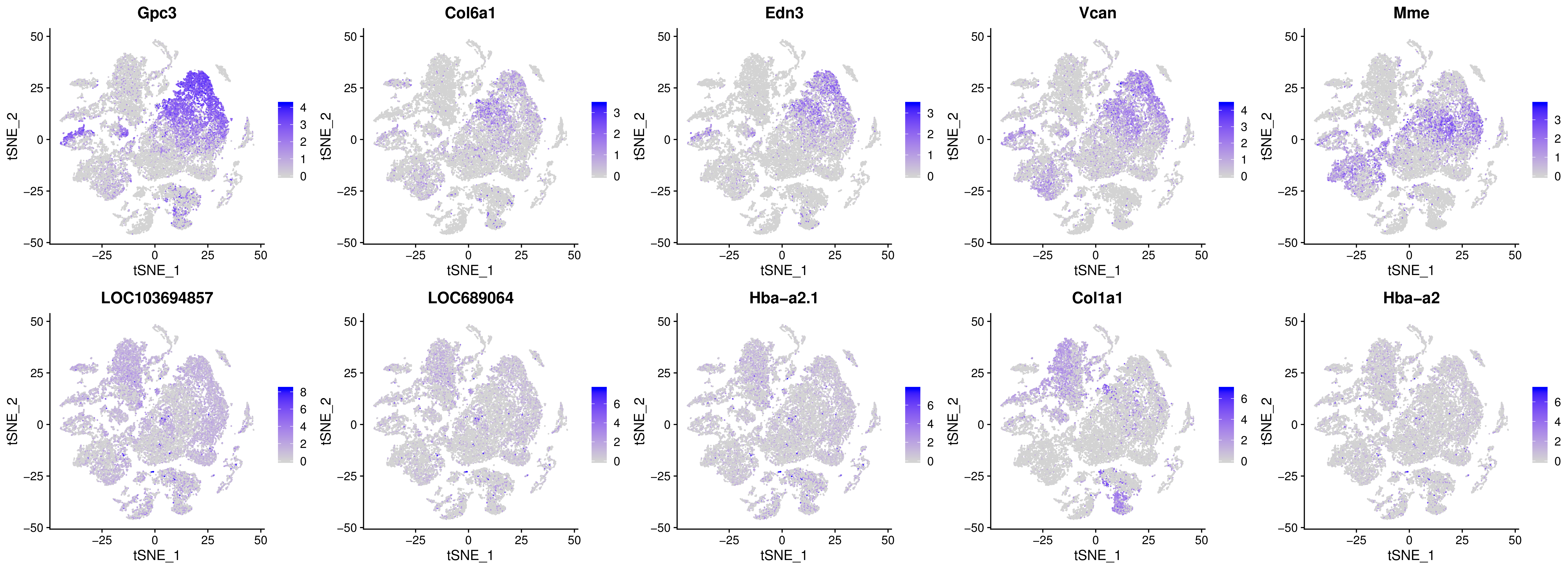

Supplement: Supplementary file 1 [file Data_Sheet_1.ZIP › S5.png]

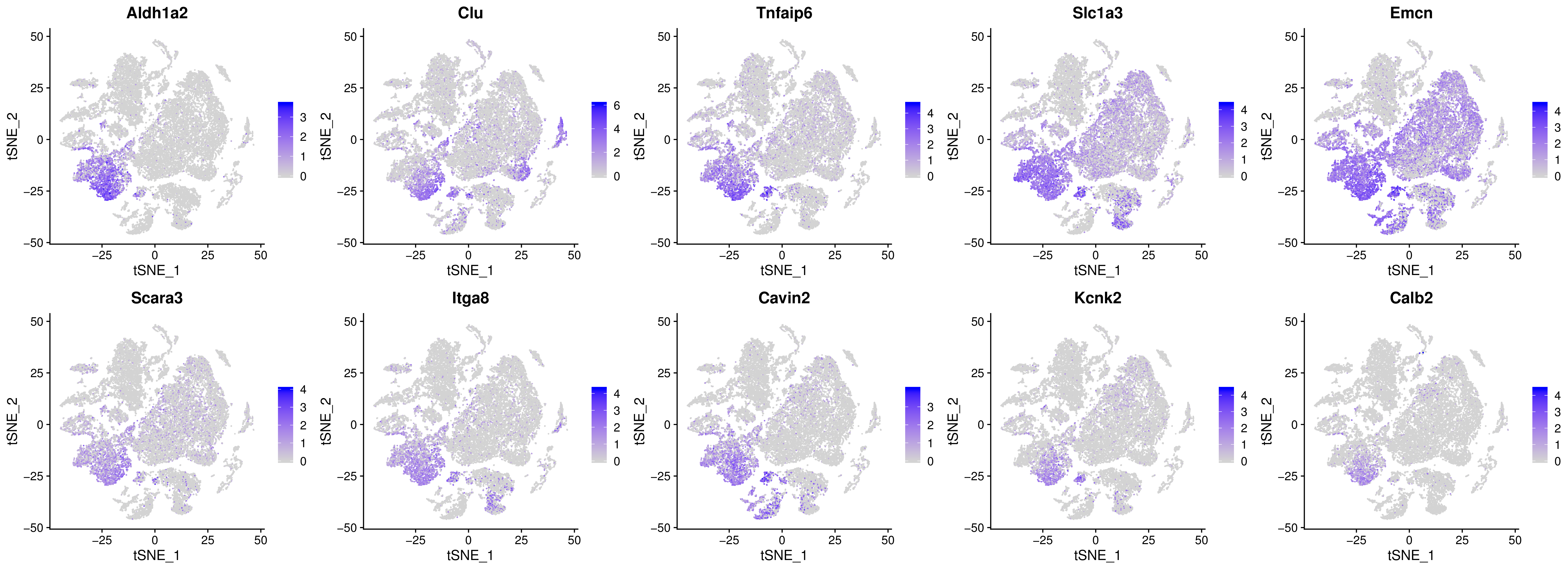

Supplement: Supplementary file 1 [file Data_Sheet_1.ZIP › S6.png]

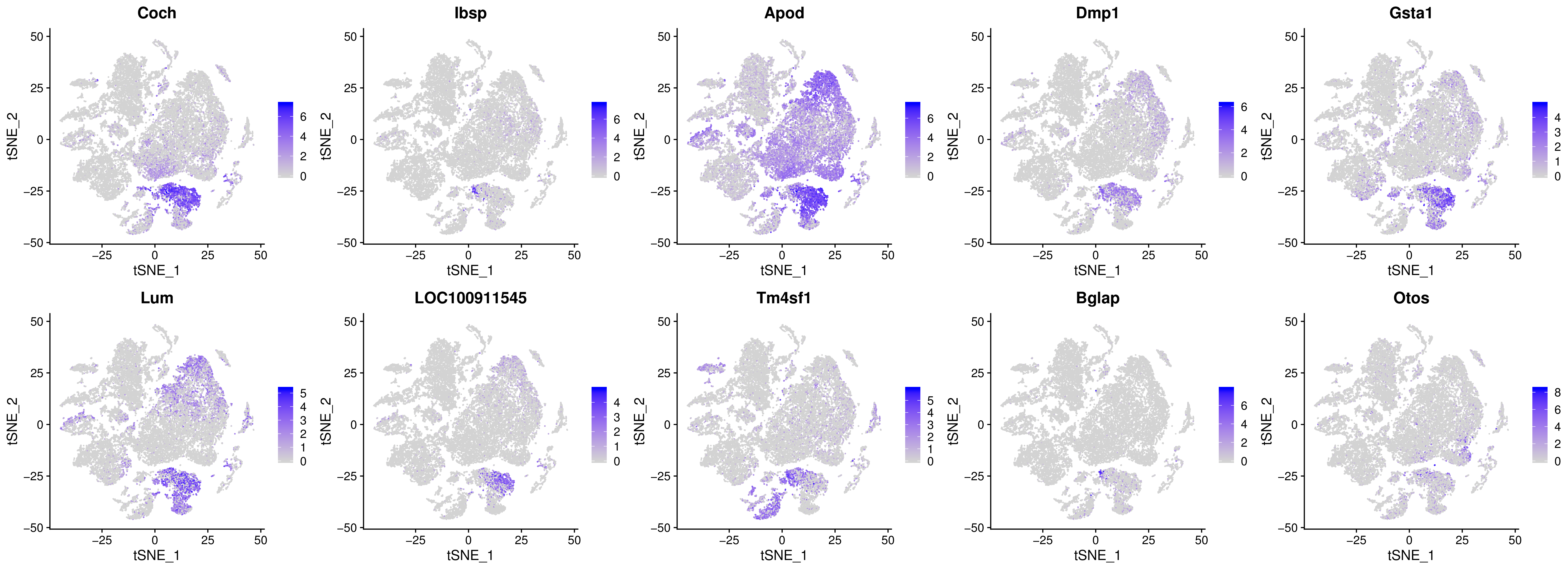

Supplement: Supplementary file 1 [file Data_Sheet_1.ZIP › S7.png]

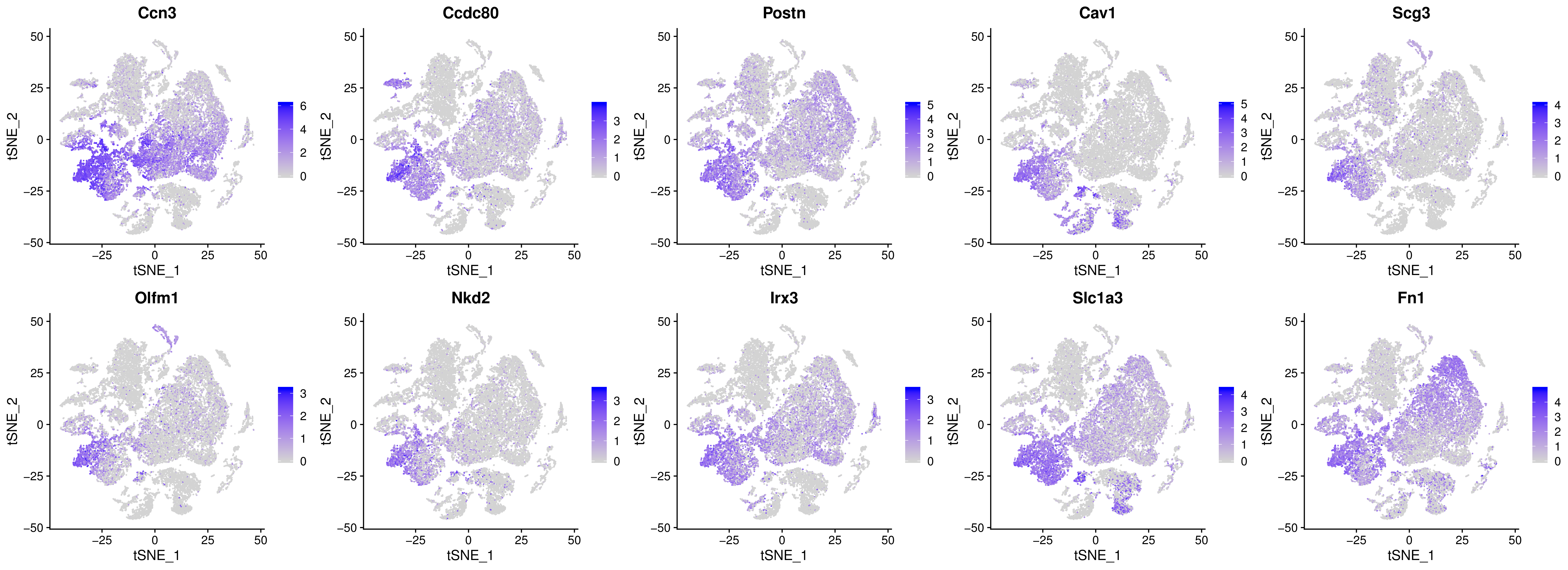

Supplement: Supplementary file 1 [file Data_Sheet_1.ZIP › S8.png]

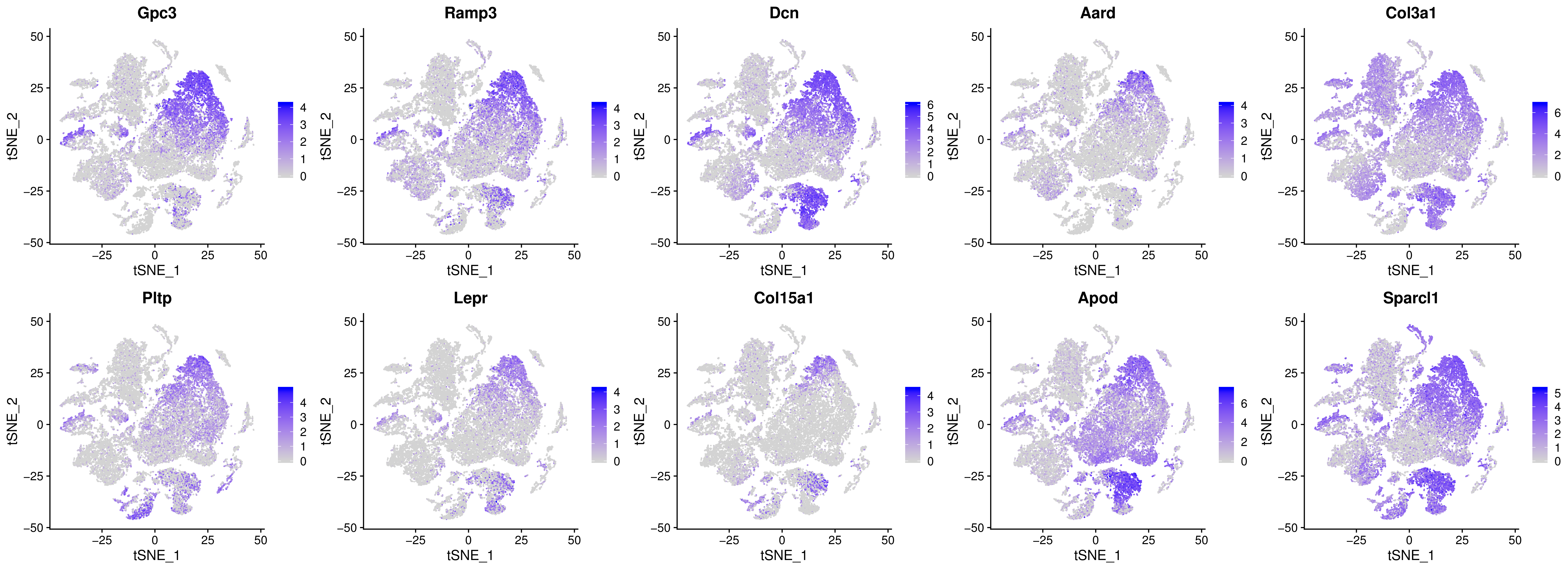

Supplement: Supplementary file 1 [file Data_Sheet_1.ZIP › S9.png]

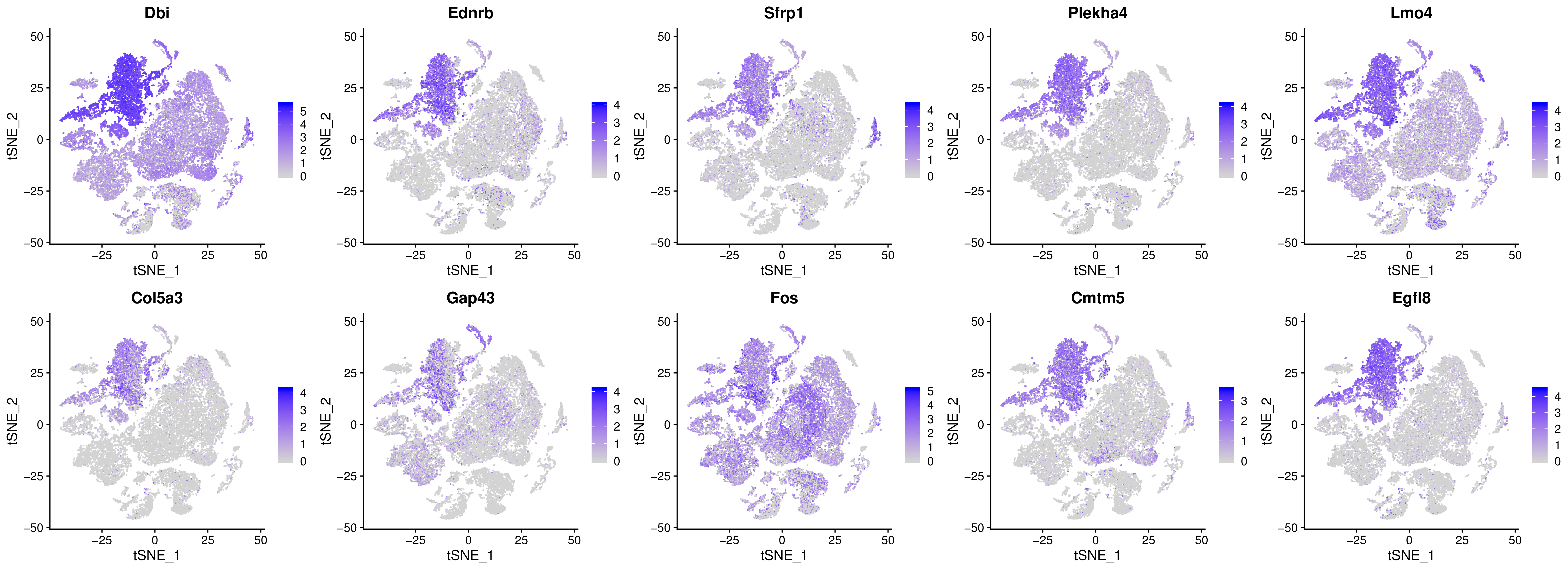

Supplement: Supplementary file 2 [file Data_Sheet_2.ZIP › S10.png]

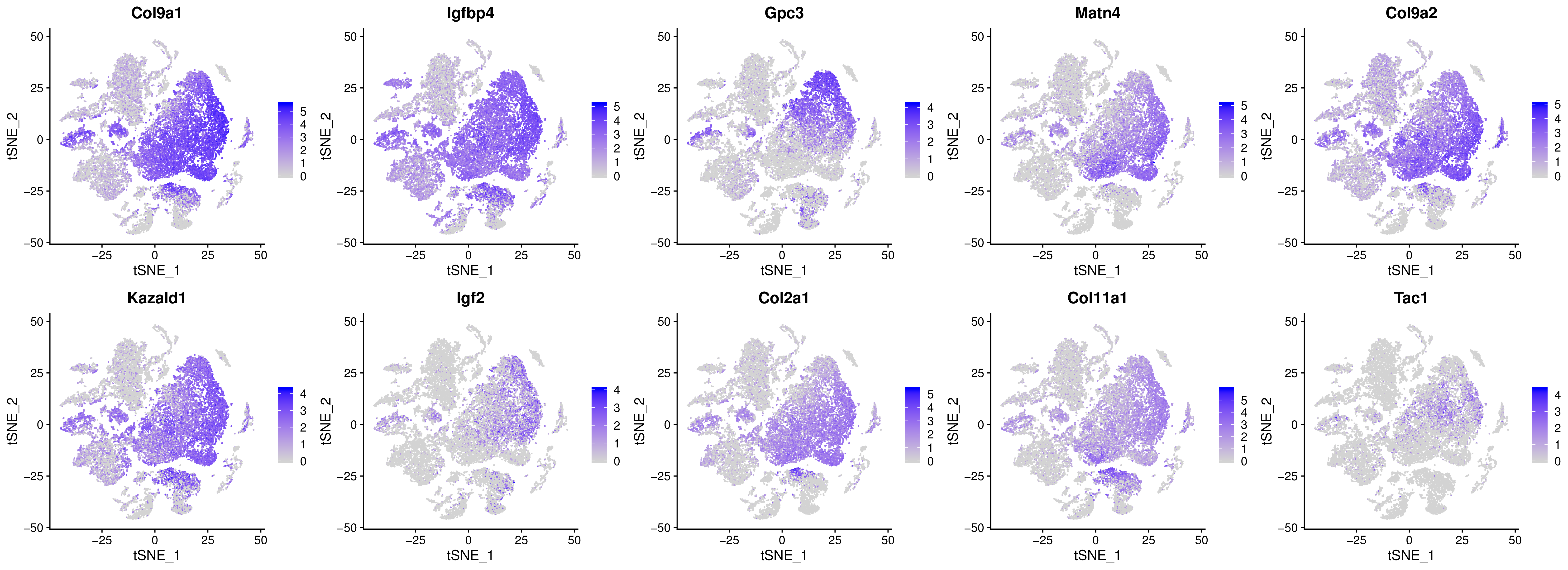

Supplement: Supplementary file 2 [file Data_Sheet_2.ZIP › S11.png]

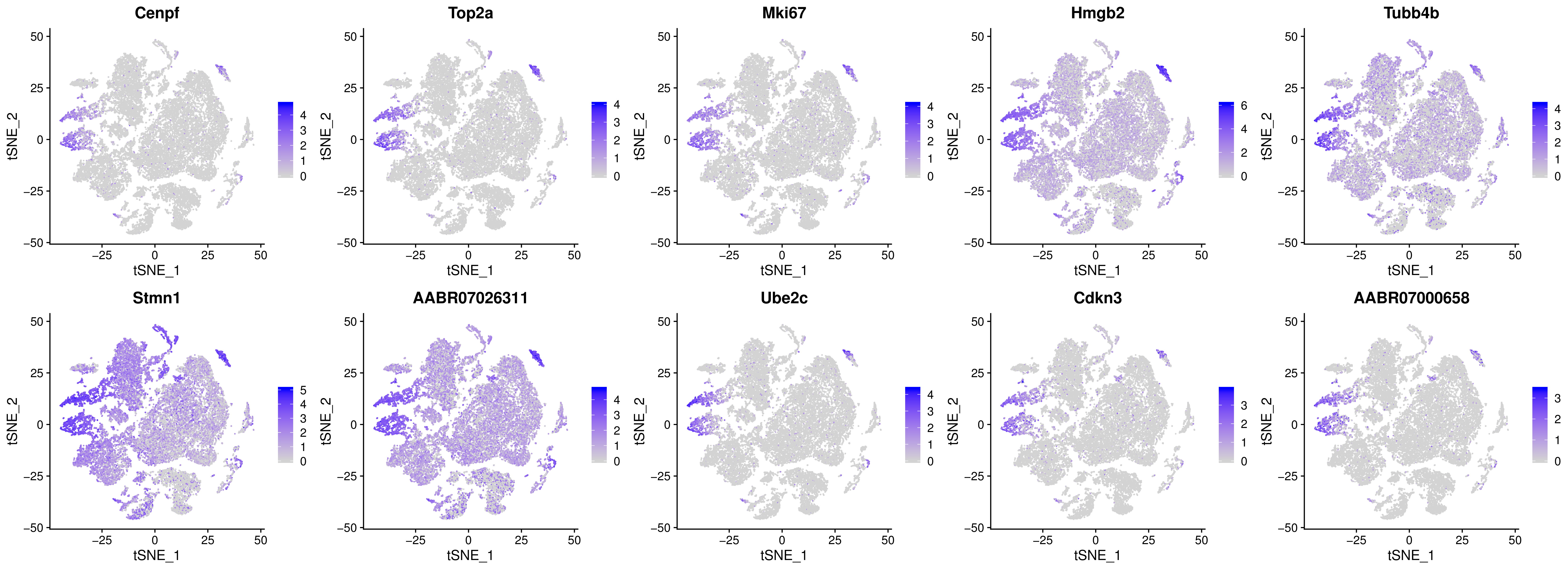

Supplement: Supplementary file 2 [file Data_Sheet_2.ZIP › S12.png]

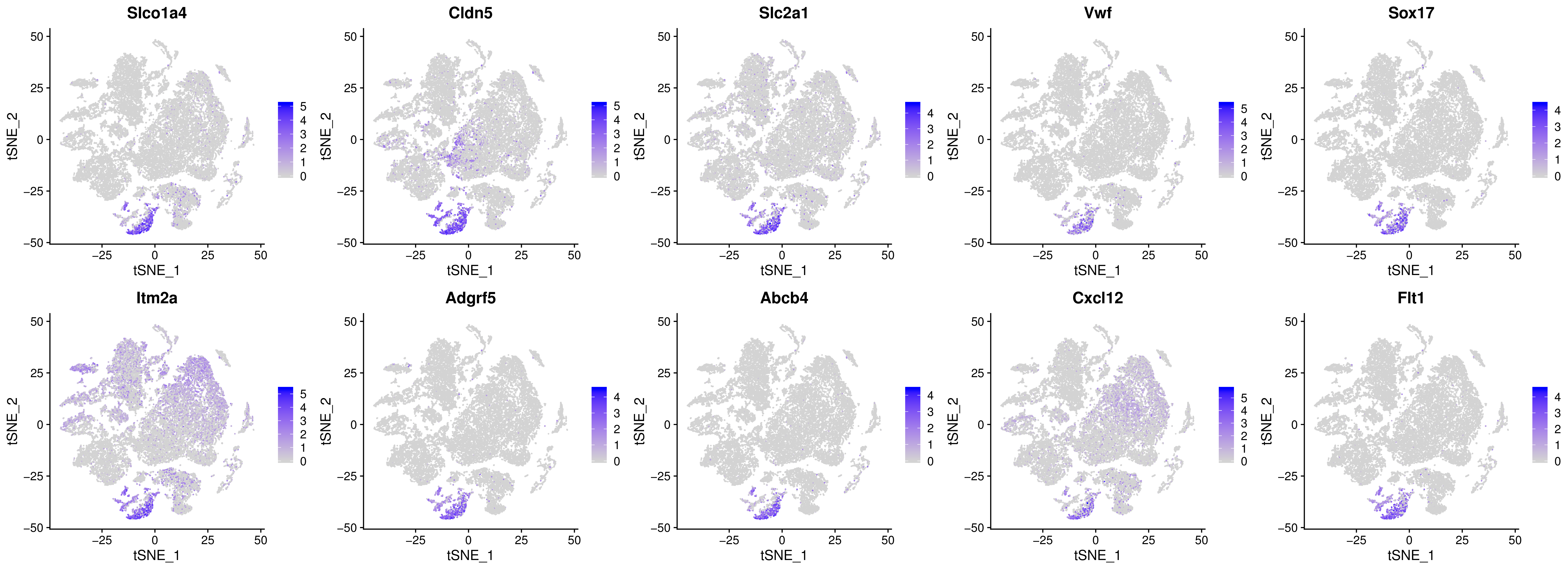

Supplement: Supplementary file 2 [file Data_Sheet_2.ZIP › S13.png]

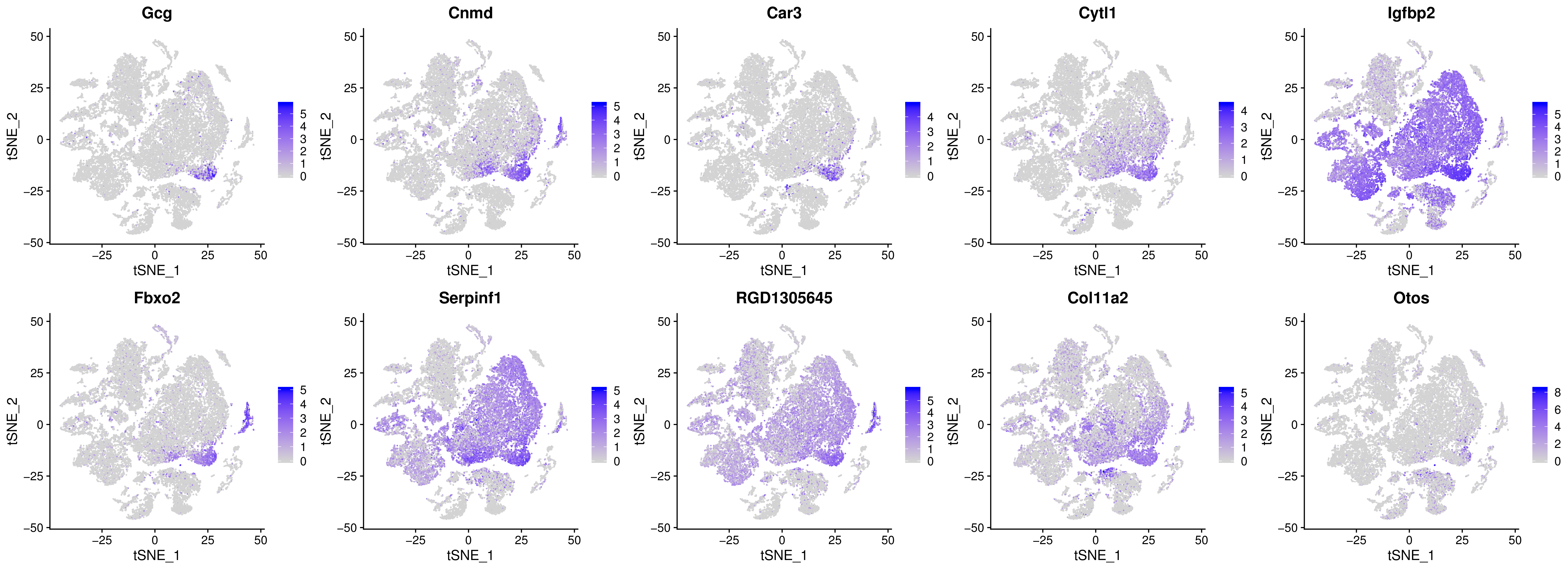

Supplement: Supplementary file 2 [file Data_Sheet_2.ZIP › S14.png]

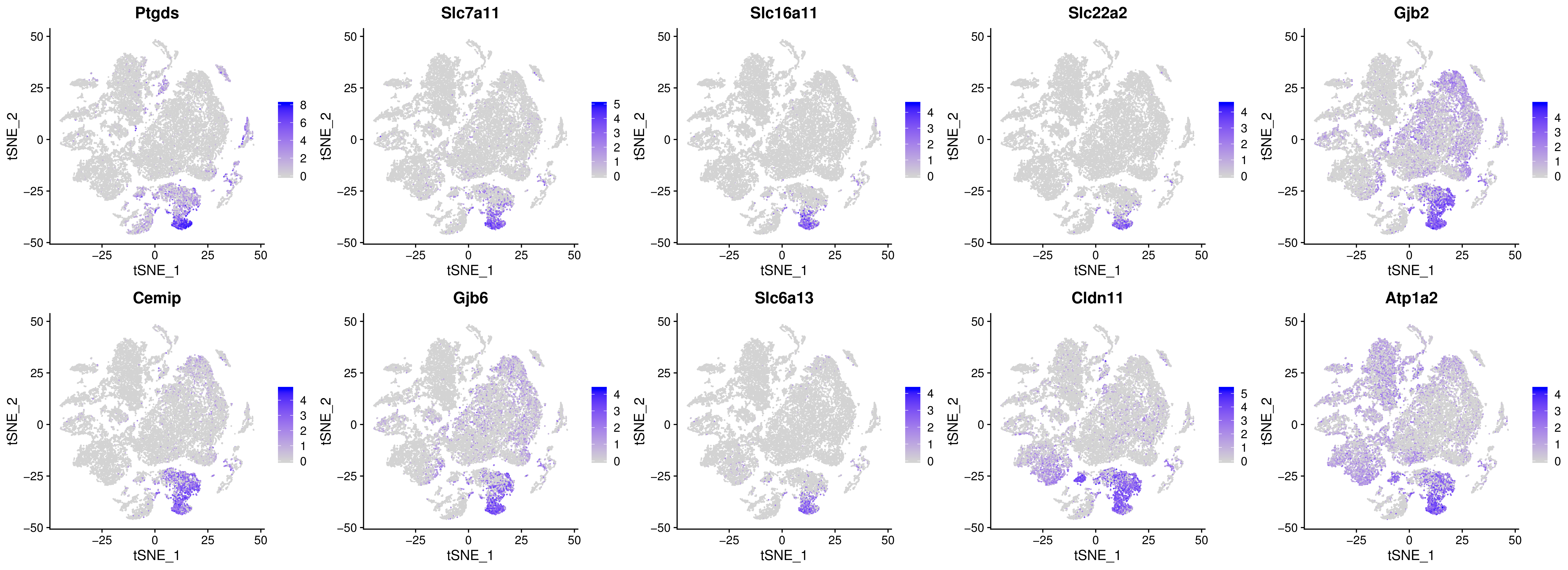

Supplement: Supplementary file 2 [file Data_Sheet_2.ZIP › S15.png]

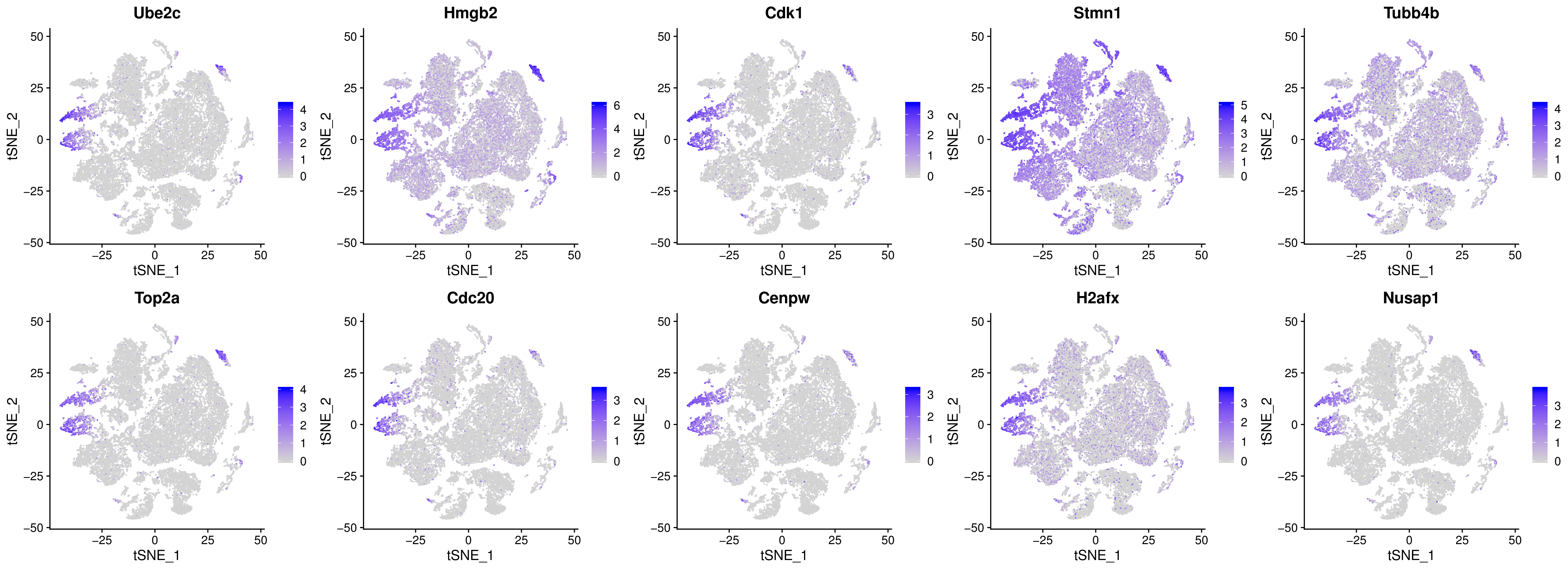

Supplement: Supplementary file 2 [file Data_Sheet_2.ZIP › S16.png]

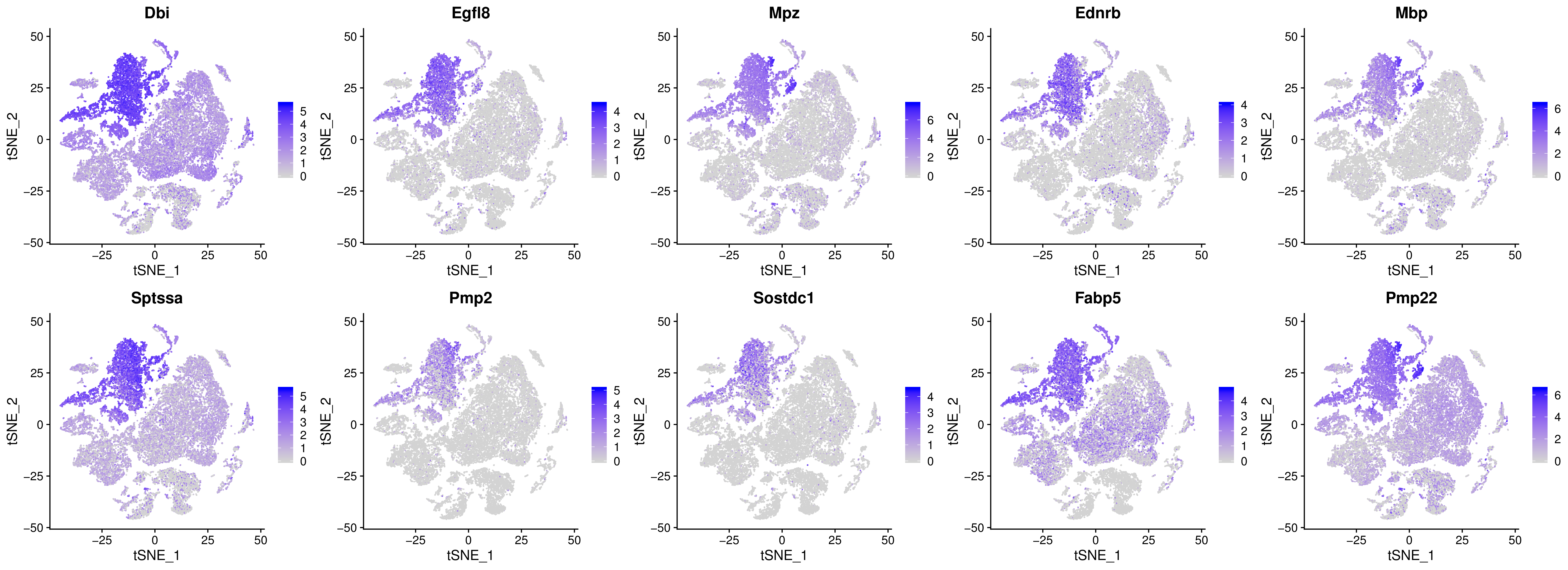

Supplement: Supplementary file 2 [file Data_Sheet_2.ZIP › S17.png]

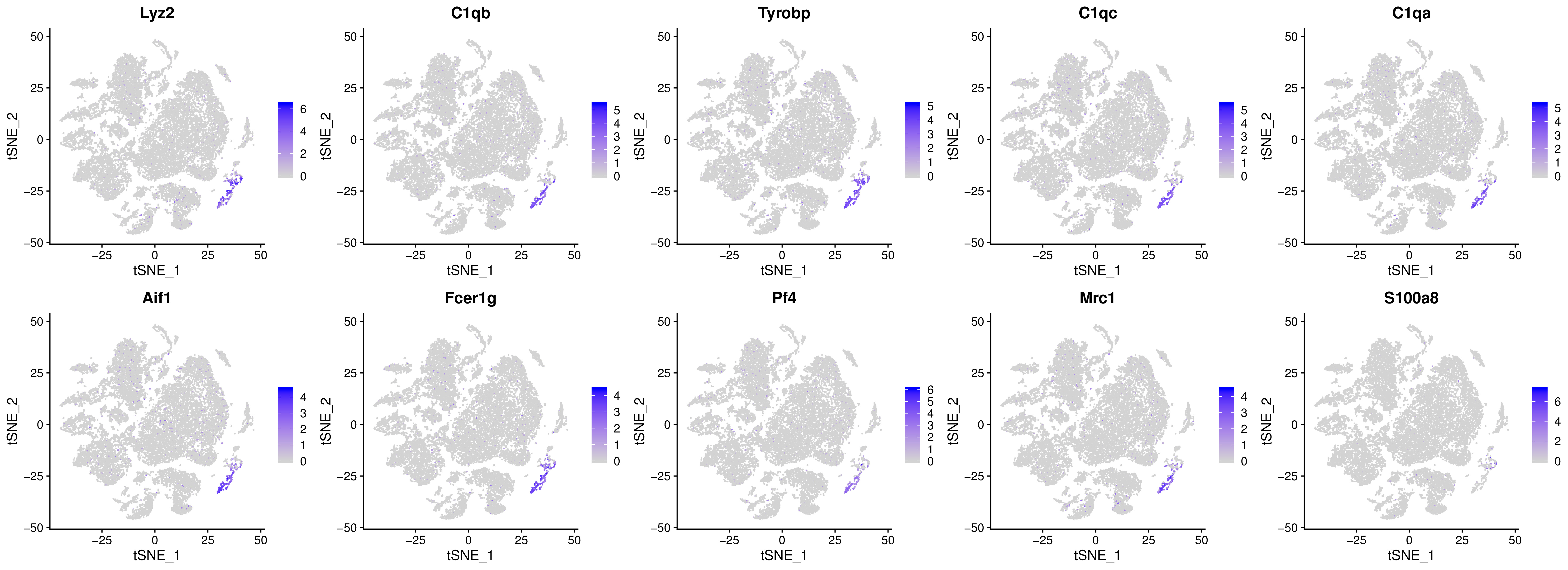

Supplement: Supplementary file 2 [file Data_Sheet_2.ZIP › S18.png]

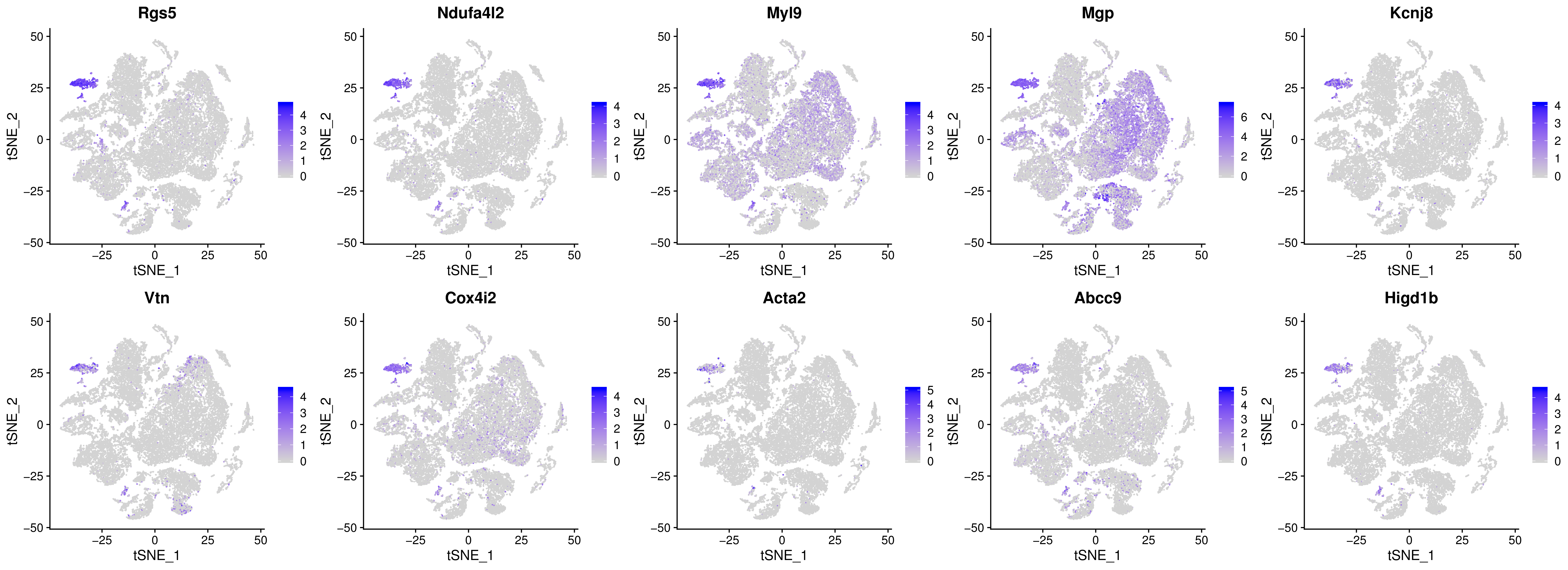

Supplement: Supplementary file 2 [file Data_Sheet_2.ZIP › S19.png]

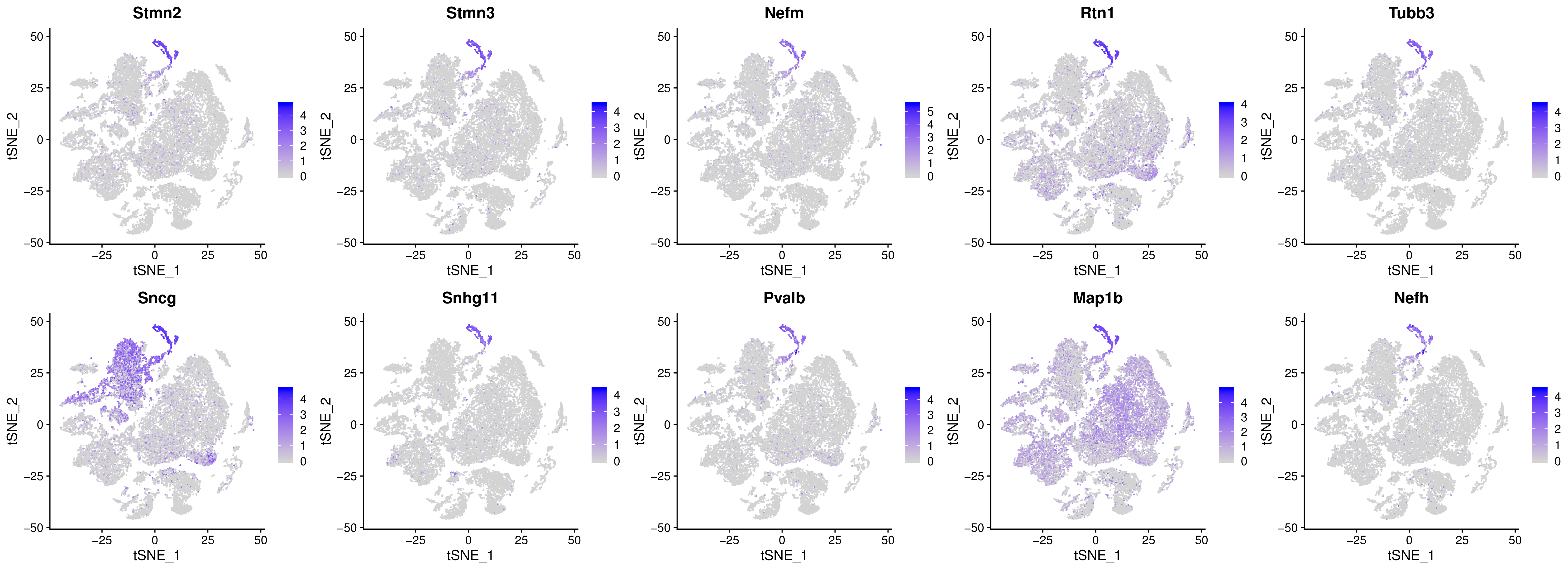

Supplement: Supplementary file 3 [file Data_Sheet_3.ZIP › S20.png]

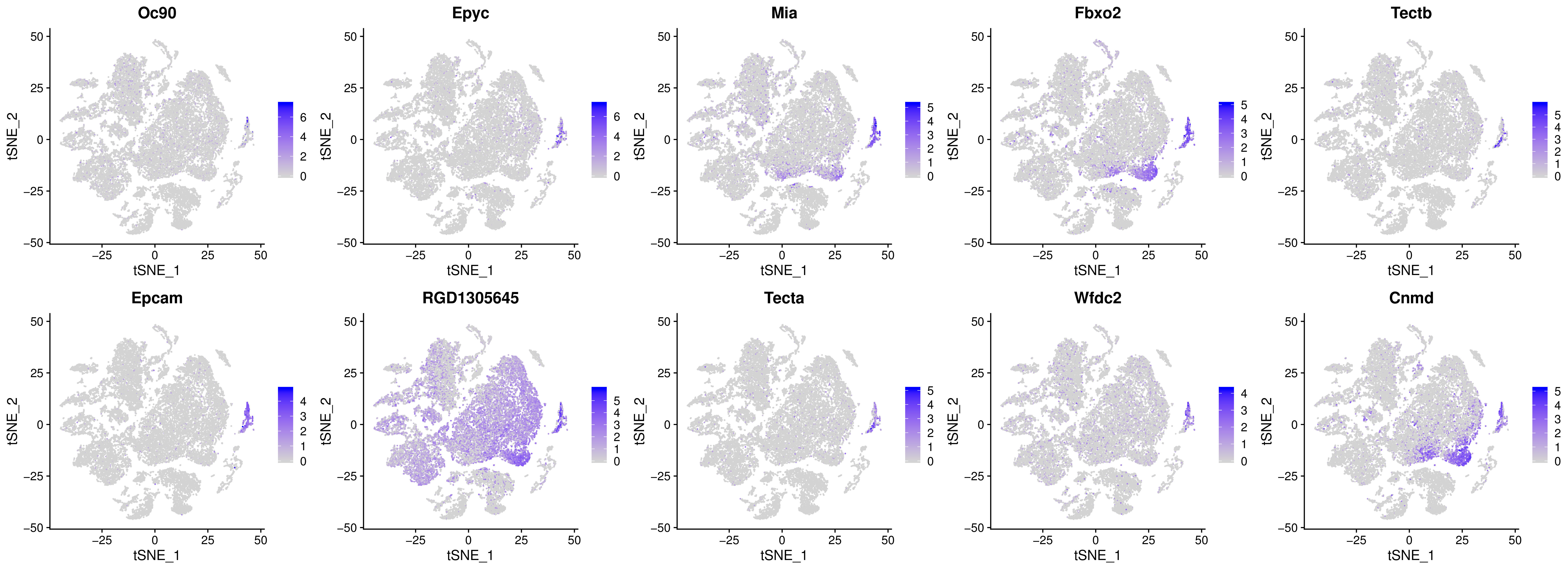

Supplement: Supplementary file 3 [file Data_Sheet_3.ZIP › S21.png]

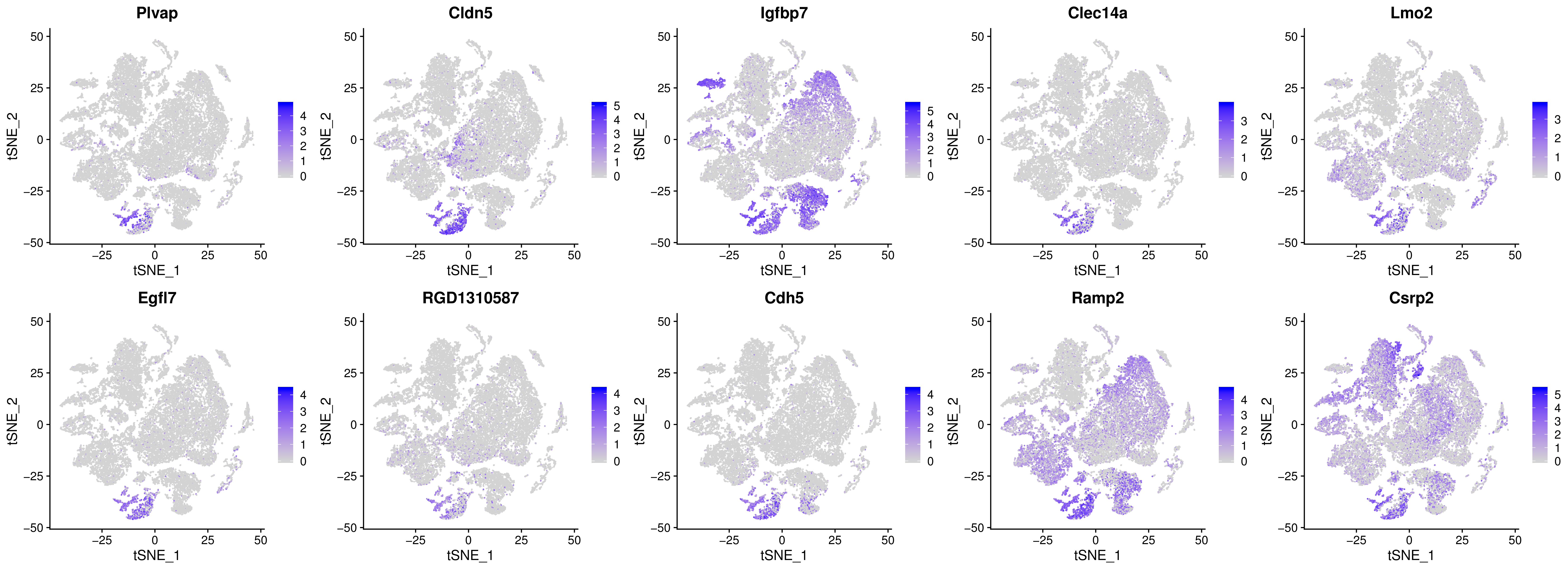

Supplement: Supplementary file 3 [file Data_Sheet_3.ZIP › S22.png]

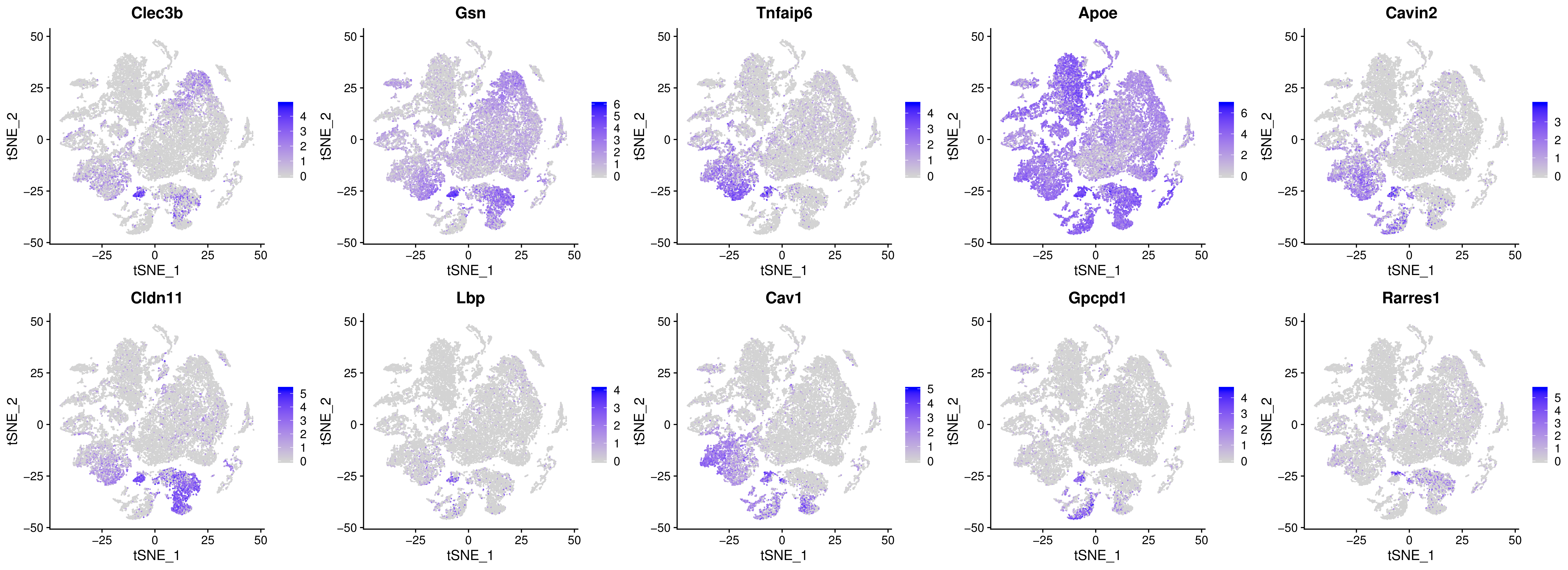

Supplement: Supplementary file 3 [file Data_Sheet_3.ZIP › S23.png]

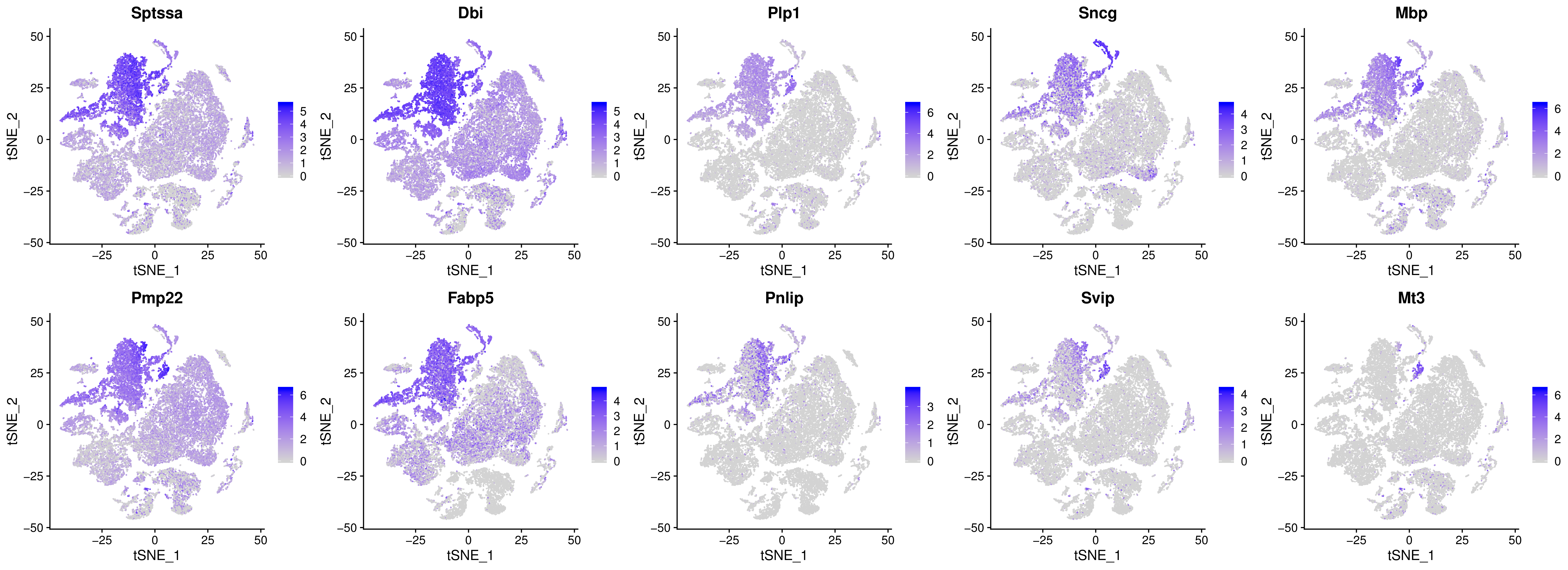

Supplement: Supplementary file 3 [file Data_Sheet_3.ZIP › S24.png]

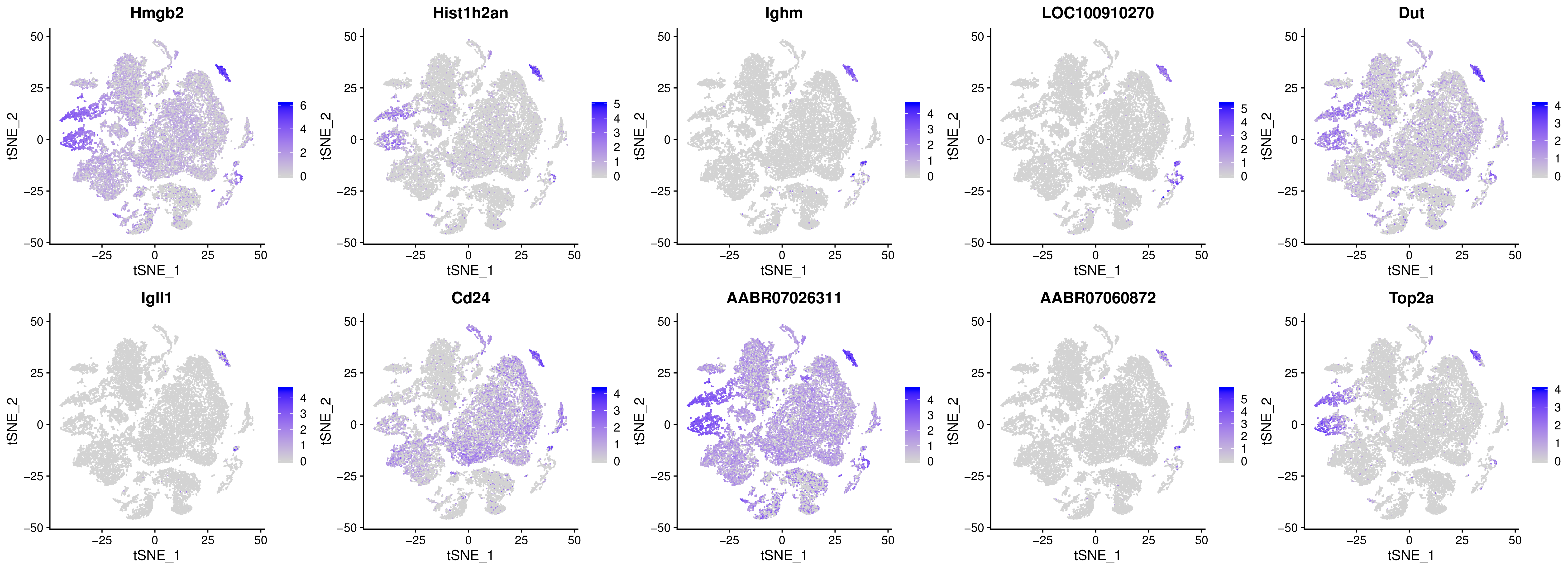

Supplement: Supplementary file 3 [file Data_Sheet_3.ZIP › S25.png]

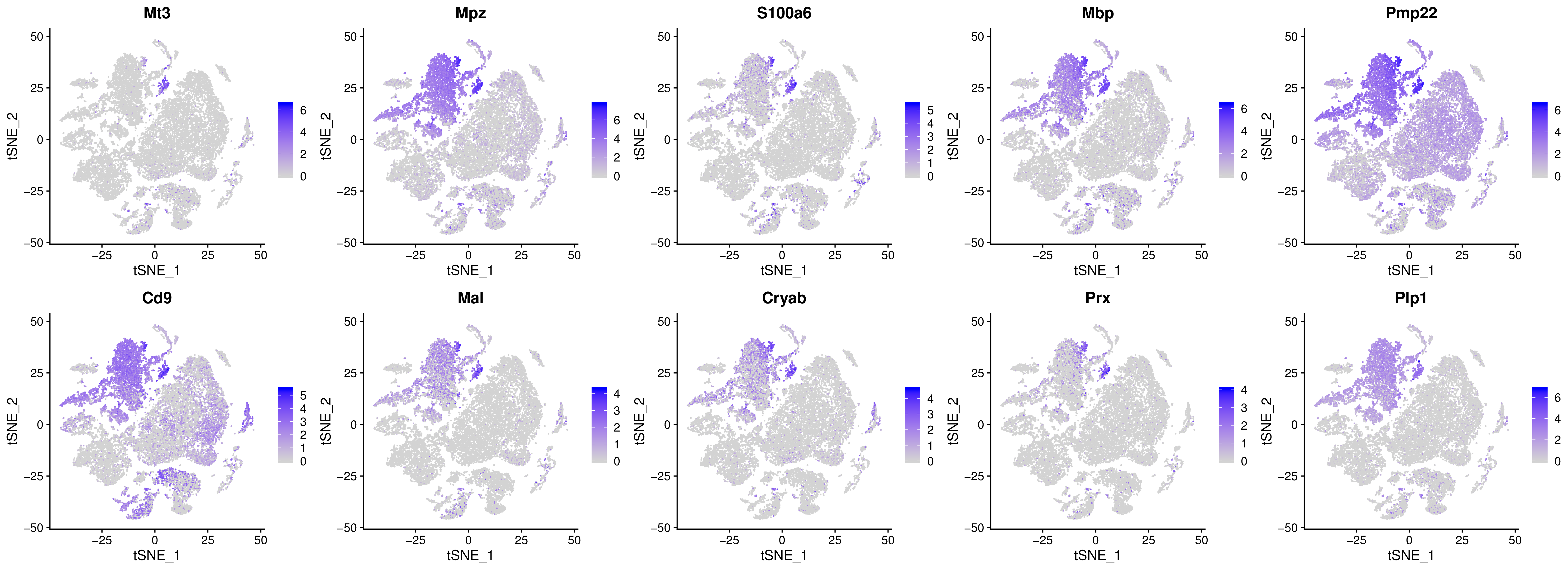

Supplement: Supplementary file 3 [file Data_Sheet_3.ZIP › S26.png]

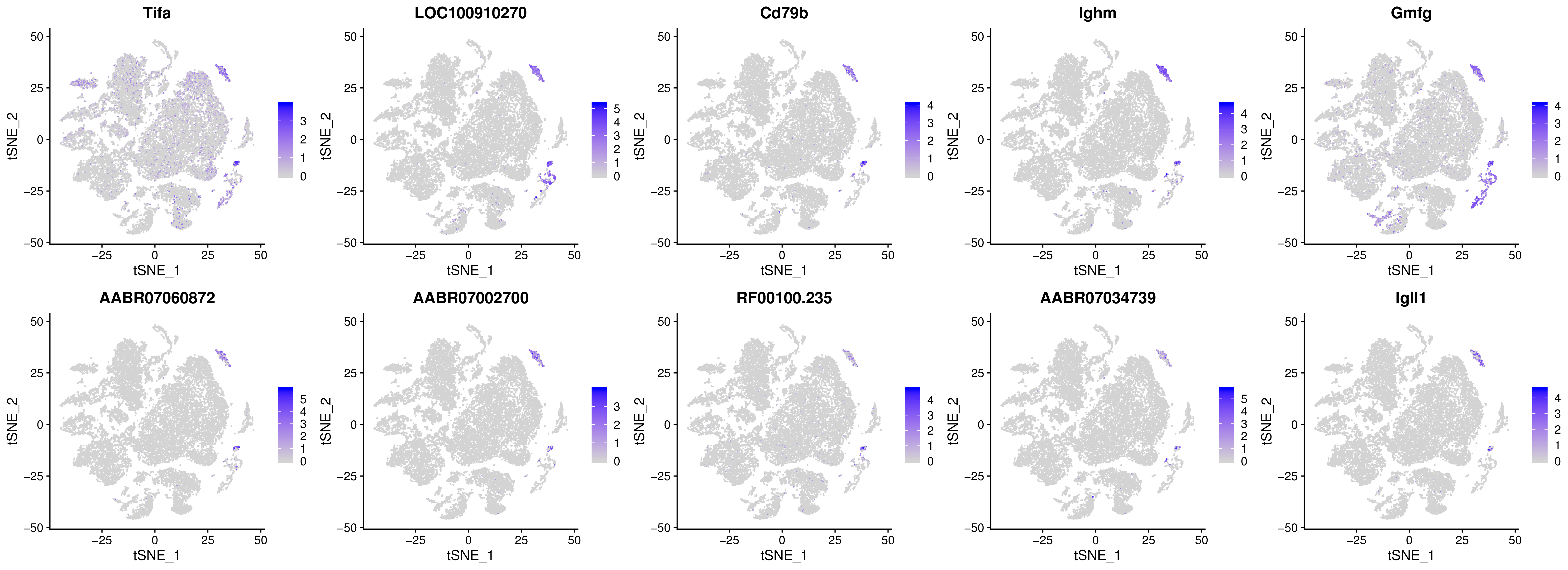

Supplement: Supplementary file 3 [file Data_Sheet_3.ZIP › S27.png]

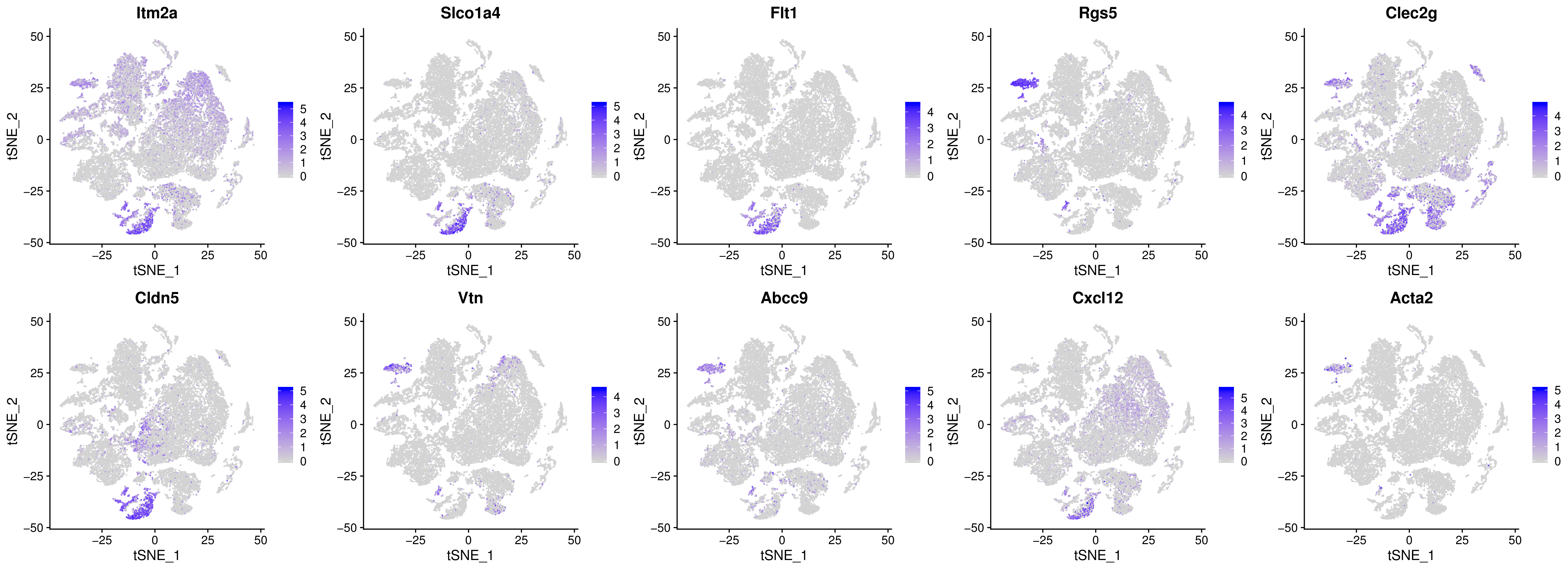

Supplement: Supplementary file 3 [file Data_Sheet_3.ZIP › S28.png]
